# Supplementary material for: Germline polymorphism in the immunoglobulin kappa and lambda loci explain variation in the expressed light chain antibody repertoire
Source: bioRxiv. 2025 Jun 1:2025.05.28.656470. Preprint. [Version 1] doi: 10.1101/2025.05.28.656470 (PMC12154815; doi:10.1101/2025.05.28.656470)
Supplement: Supplementary file 1 [file media-1.pdf]

Figure S1

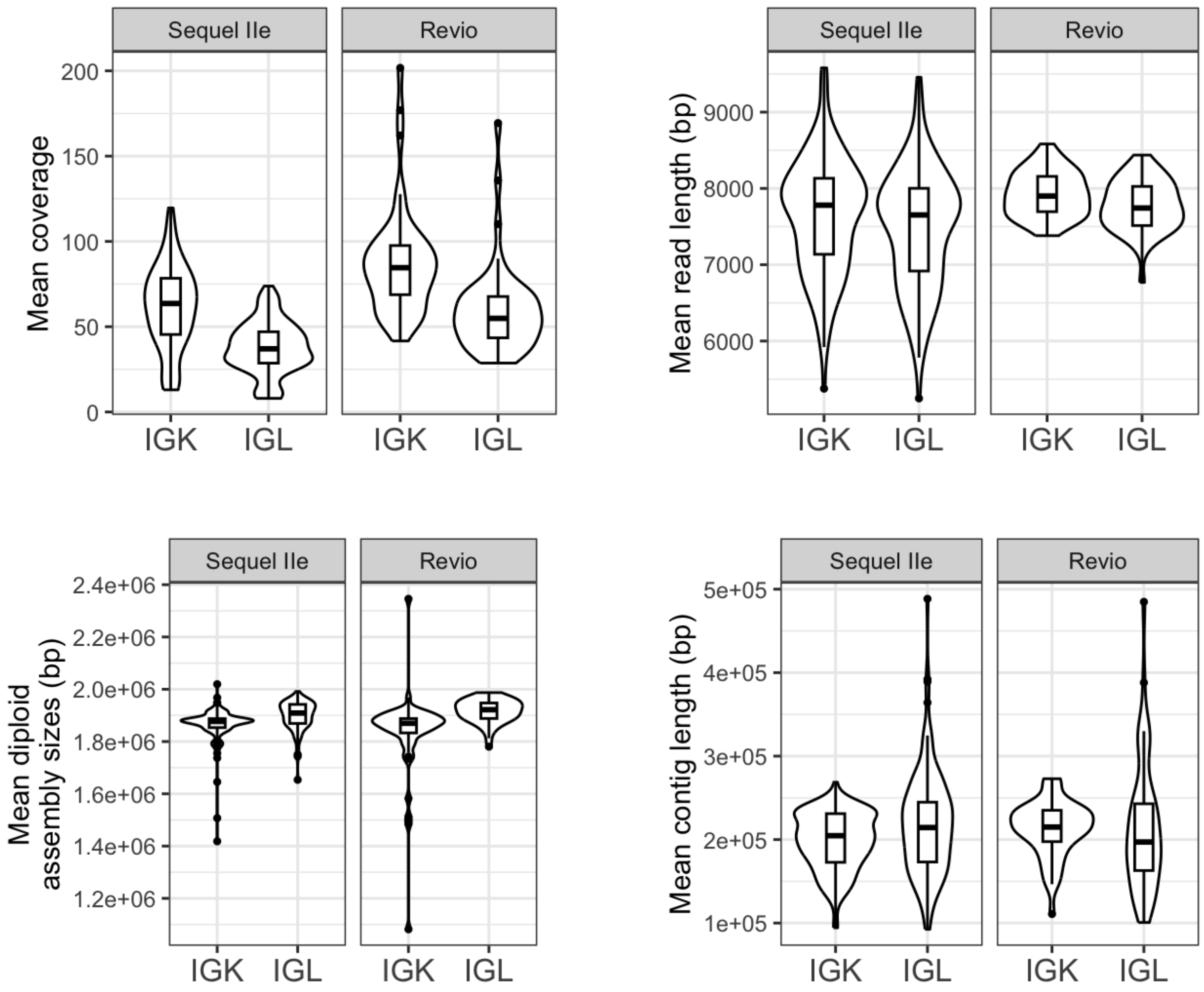
Sequel IIe
Revio
Mean coverage
200
150
100
50
0
IGK
IGL
Mean read length (bp)
9000
8000
7000
6000
Mean diploid assembly sizes (bp)
2.4e+06
2.2e+06
2.0e+06
1.8e+06
1.6e+06
1.4e+06
1.2e+06
Mean contig length (bp)
5e+05
4e+05
3e+05
2e+05
1e+05

Figure S2

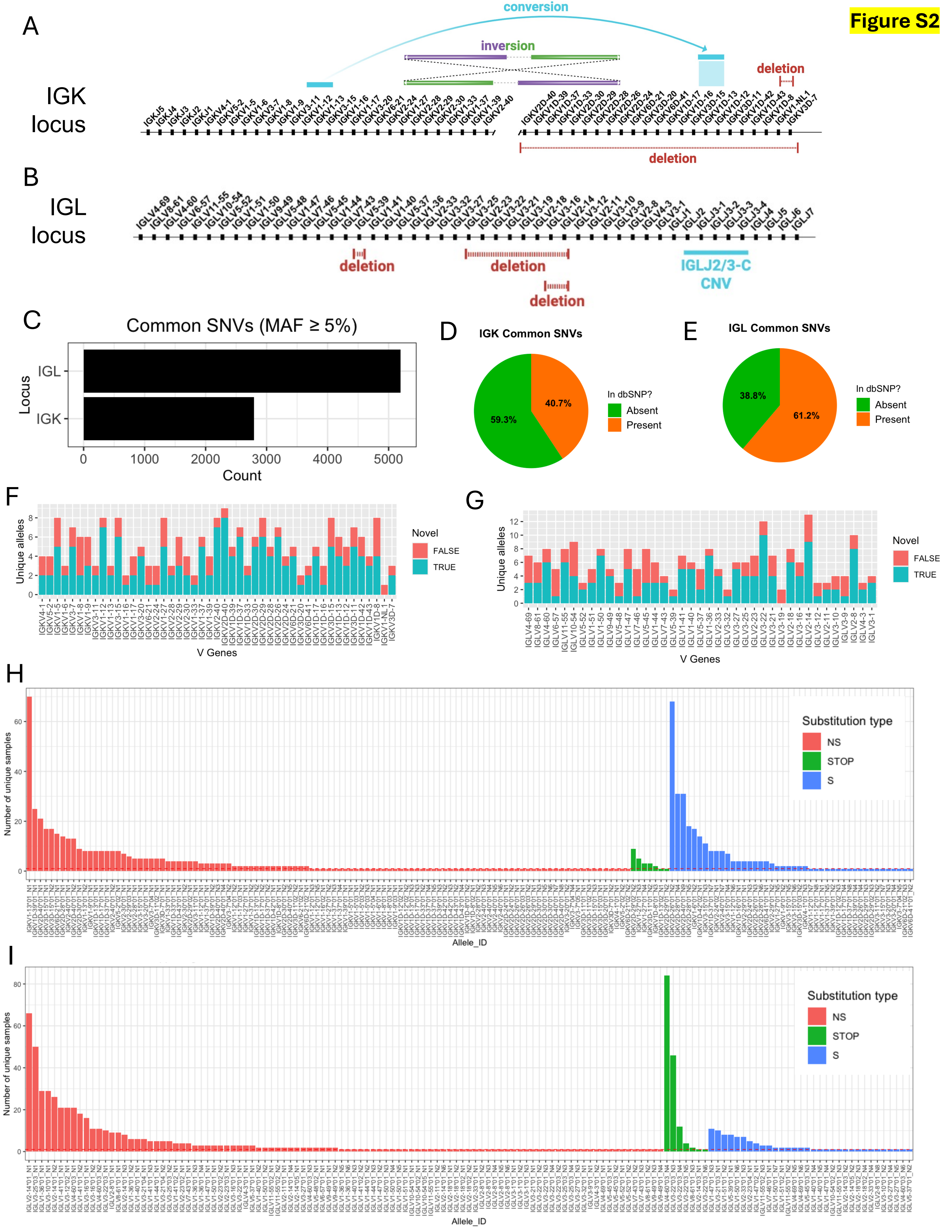
A
conversion
inversion
deletion
IGK locus
IGKJ5 IGKJ4 IGKJ3 IGKJ2 IGKJ1 IGKV4-1 IGKV5-2 IGKV1-5 IGKV1-6 IGKV3-7 IGKV1-8 IGKV1-9 IGKV3-11 IGKV1-12 IGKV1-13 IGKV3-15 IGKV1-16 IGKV1-17 IGKV3-20 IGKV6-21 IGKV2-24 IGKV1-27 IGKV2-28 IGKV2-29 IGKV2-30 IGKV1-33 IGKV1-37 IGKV1-39 IGKV2-40
IGKV2D-40 IGKV1D-39 IGKV1D-37 IGKV1D-33 IGKV2D-30 IGKV2D-29 IGKV2D-28 IGKV2D-26 IGKV2D-24 IGKV6D-21 IGKV3D-20 IGKV6D-41 IGKV1D-17 IGKV1D-16 IGKV3D-15 IGKV1D-13 IGKV1D-12 IGKV3D-11 IGKV1D-42 IGKV1D-43 IGKV1D-8 IGKV1-NL1 IGKV3D-7
deletion
B
IGL locus
IGLV4-69 IGLV8-61 IGLV4-60 IGLV6-57 IGLV11-55 IGLV10-54 IGLV5-52 IGLV1-51 IGLV1-50 IGLV9-49 IGLV5-48 IGLV1-47 IGLV7-46 IGLV5-45 IGLV1-44 IGLV7-43 IGLV5-39 IGLV1-41 IGLV1-40 IGLV5-37 IGLV1-36 IGLV2-33 IGLV3-32 IGLV3-27 IGLV3-25 IGLV2-23 IGLV3-22 IGLV3-21 IGLV3-19 IGLV2-18 IGLV3-16 IGLV2-14 IGLV3-12 IGLV2-11 IGLV3-10 IGLV3-9 IGLV2-8 IGLV4-3 IGLV3-1 IGLJ1 IGLJ2 IGLJ3-1 IGLJ3-2 IGLJ3-3 IGLJ3-4 IGLJ4 IGLJ5 IGLJ6 IGLJ7
deletion
deletion
deletion
IGLJ2/3-C CNV
C
Common SNVs (MAF ≥ 5%)
Locus
IGL
IGK
0 1000 2000 3000 4000 5000
Count
D
IGK Common SNVs
40.7%
59.3%
In dbSNP?
Absent
Present
E
IGL Common SNVs
38.8%
61.2%
In dbSNP?
Absent
Present
F
Unique alleles
V Genes
Novel
FALSE
TRUE
G
Unique alleles
V Genes
Novel
FALSE
TRUE
H
Number of unique samples
Allele_ID
Substitution type
NS
STOP
S
I
Number of unique samples
Allele_ID
Substitution type
NS
STOP
S

Figure S3

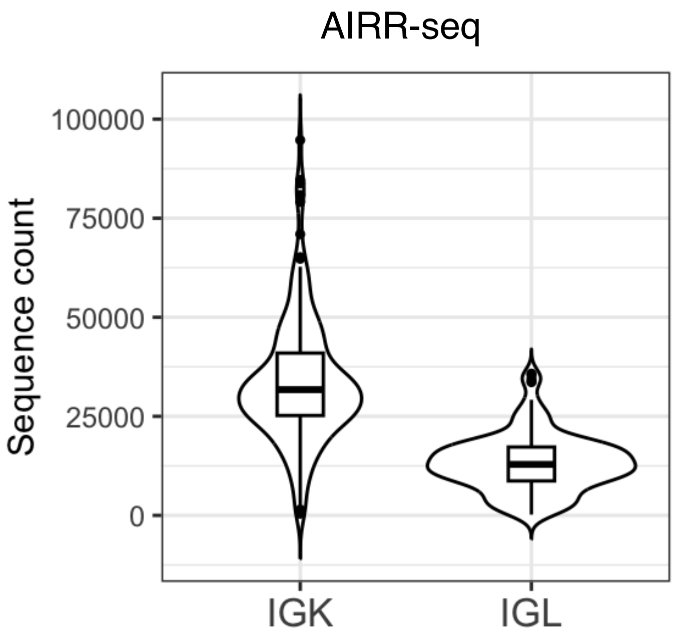

Figure S4

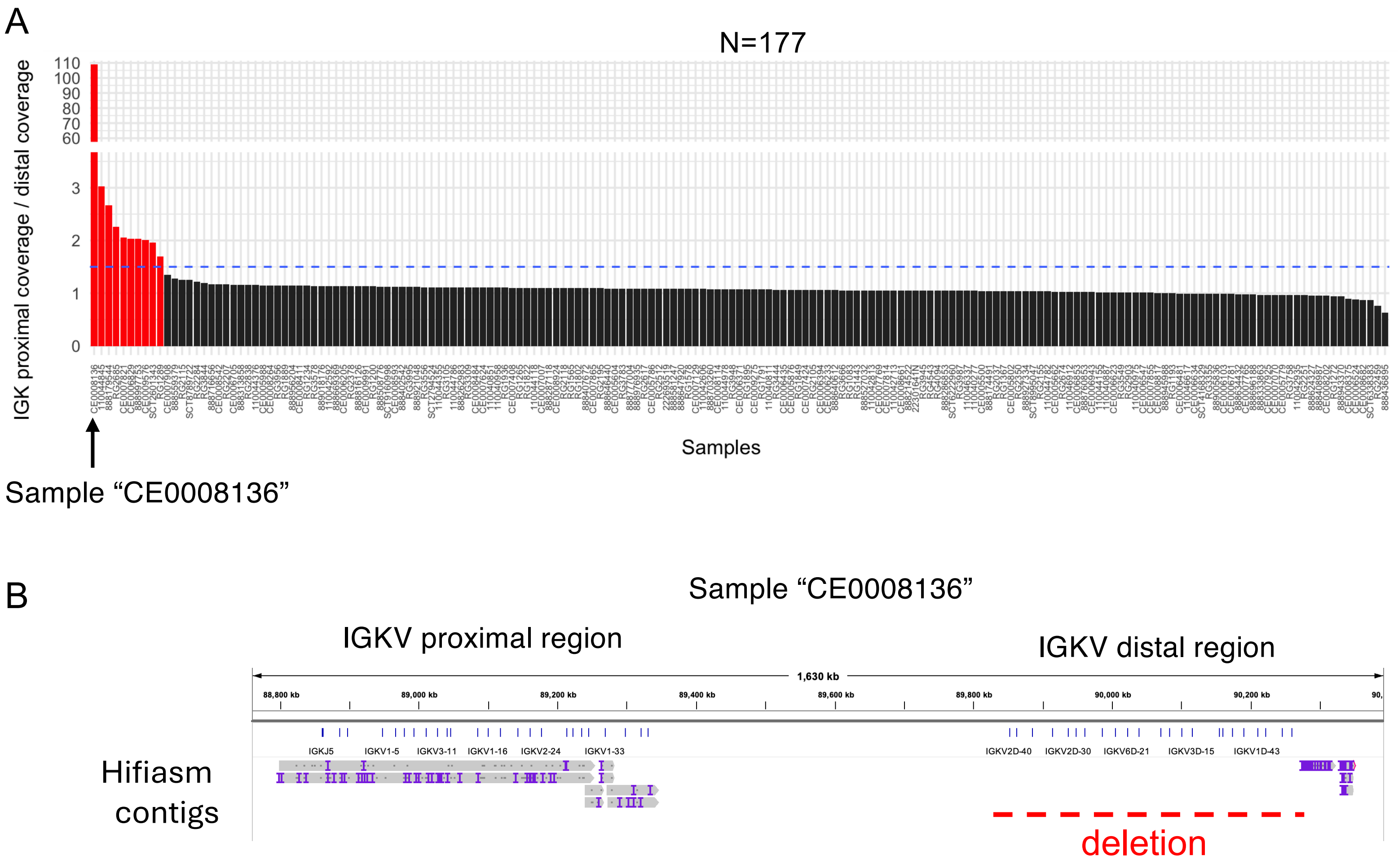
A
N=177
IGK proximal coverage / distal coverage
110
100
90
80
70
60
3
2
1
0
Samples
Sample "CE0008136"
B
Sample "CE0008136"
IGKV proximal region
IGKV distal region
1,630 kb
88,800 kb
89,000 kb
89,200 kb
89,400 kb
89,600 kb
89,800 kb
90,000 kb
90,200 kb
IGKJ5
IGKV1-5
IGKV3-11
IGKV1-16
IGKV2-24
IGKV1-33
IGKV2D-40
IGKV2D-30
IGKV6D-21
IGKV3D-15
IGKV1D-43
Hifiasm contigs
deletion

Figure S5

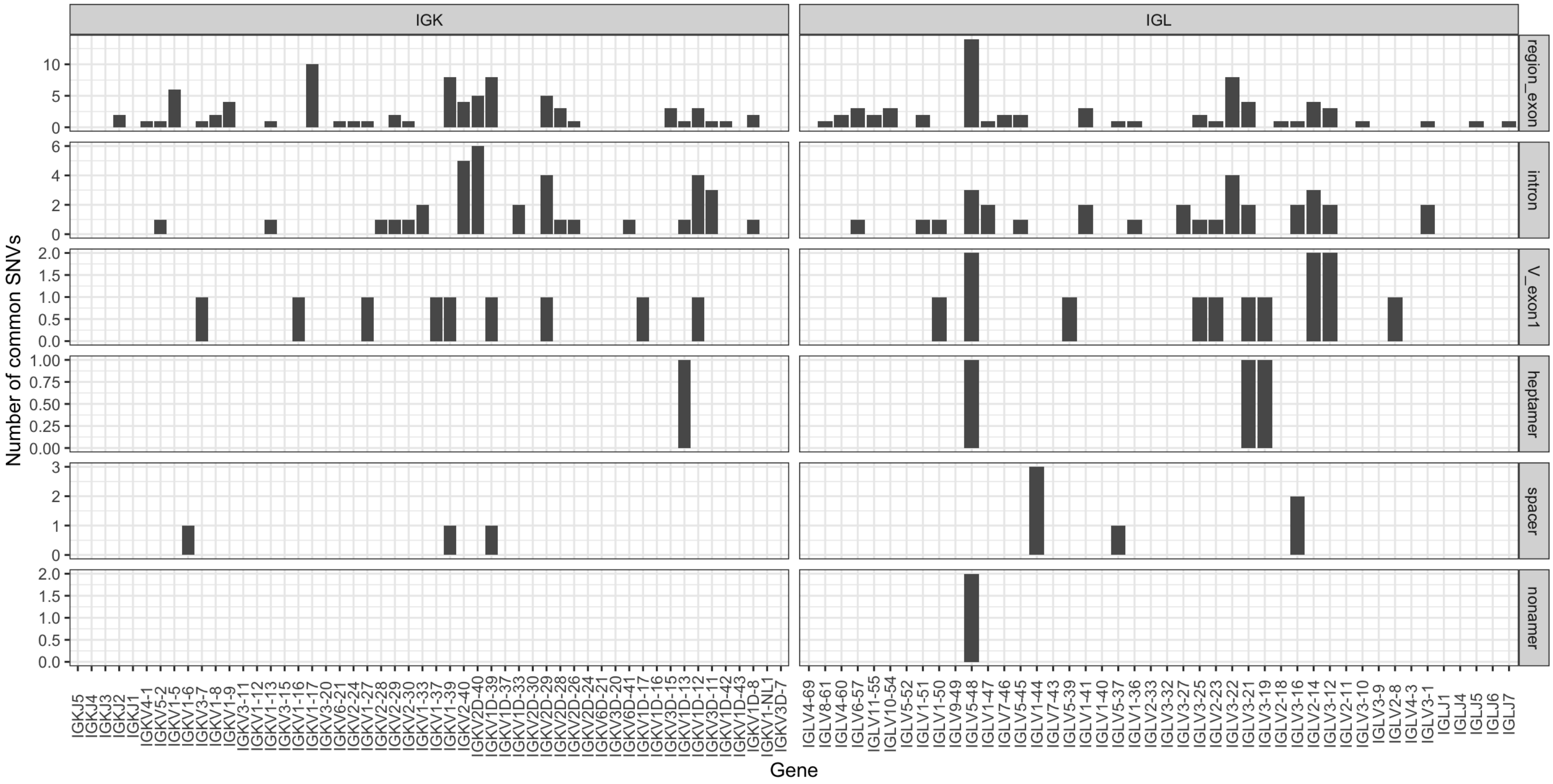
IGK
IGL
region_exon
intron
V_exon1
heptamer
spacer
nonamer
Number of common SNVs
Gene
IGKJ5 IGKJ4 IGKJ3 IGKJ2 IGKJ1 IGKV4-1 IGKV5-2 IGKV1-5 IGKV1-6 IGKV3-7 IGKV1-8 IGKV1-9 IGKV3-11 IGKV1-12 IGKV1-13 IGKV3-15 IGKV1-16 IGKV1-17 IGKV3-20 IGKV6-21 IGKV2-24 IGKV1-27 IGKV2-28 IGKV2-29 IGKV2-30 IGKV1-33 IGKV1-37 IGKV1-39 IGKV2-40 IGKV2D-40 IGKV1D-39 IGKV1D-37 IGKV1D-33 IGKV2D-30 IGKV2D-29 IGKV2D-28 IGKV2D-26 IGKV2D-24 IGKV6D-21 IGKV3D-20 IGKV6D-41 IGKV1D-17 IGKV1D-16 IGKV3D-15 IGKV1D-13 IGKV1D-12 IGKV3D-11 IGKV1D-42 IGKV1D-43 IGKV1D-8 IGKV1-NL1 IGKV3D-7
IGLV4-69 IGLV8-61 IGLV4-60 IGLV6-57 IGLV11-55 IGLV10-54 IGLV5-52 IGLV1-51 IGLV1-50 IGLV9-49 IGLV5-48 IGLV1-47 IGLV7-46 IGLV5-45 IGLV1-44 IGLV7-43 IGLV5-39 IGLV1-41 IGLV1-40 IGLV5-37 IGLV1-36 IGLV2-33 IGLV3-32 IGLV3-27 IGLV3-25 IGLV2-23 IGLV3-22 IGLV3-21 IGLV3-19 IGLV2-18 IGLV3-16 IGLV2-14 IGLV3-12 IGLV2-11 IGLV3-10 IGLV3-9 IGLV2-8 IGLV4-3 IGLV3-1 IGLJ1 IGLJ4 IGLJ5 IGLJ6 IGLJ7

Figure S6

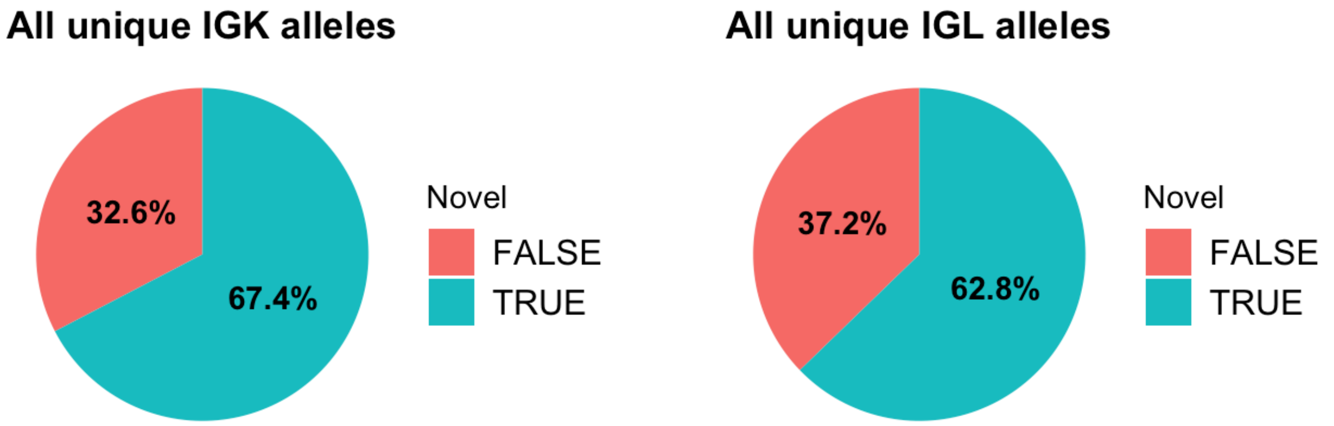

All unique IGK alleles
32.6%
67.4%
Novel
FALSE
TRUE
All unique IGL alleles
37.2%
62.8%
Novel
FALSE
TRUE

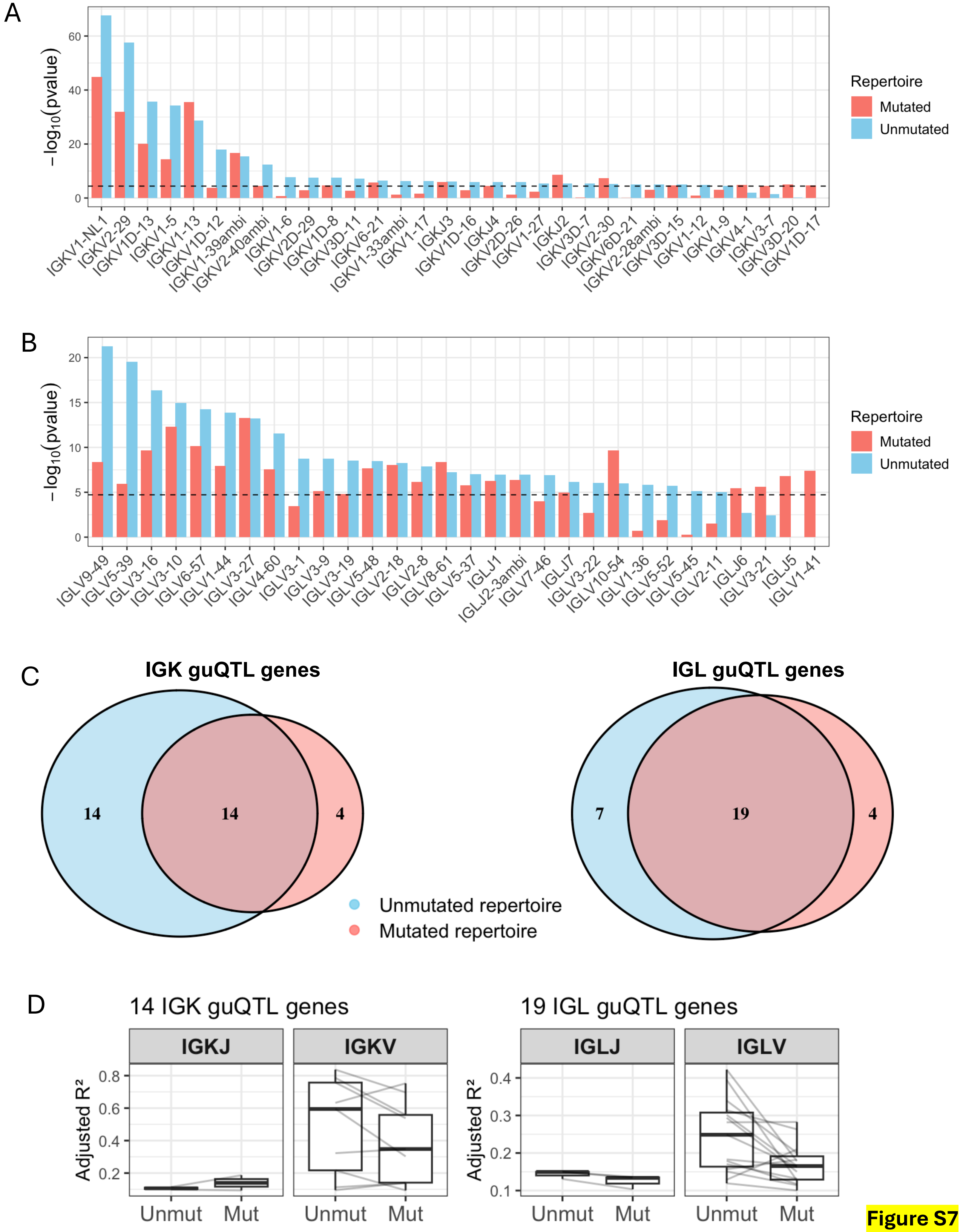

A
−log10(pvalue)
60
40
20
0
IGKV1-NL1
IGKV2-29
IGKV1D-13
IGKV1-5
IGKV1-13
IGKV1D-12
IGKV1-39ambi
IGKV2-40ambi
IGKV1-6
IGKV2D-29
IGKV1D-8
IGKV3D-11
IGKV6-21
IGKV1-33ambi
IGKV1-17
IGKJ3
IGKV1D-16
IGKJ4
IGKV2D-26
IGKV1-27
IGKJ2
IGKV3D-7
IGKV2-30
IGKV6D-21
IGKV2-28ambi
IGKV3D-15
IGKV1-12
IGKV1-9
IGKV4-1
IGKV3-7
IGKV3D-20
IGKV1D-17
Repertoire
Mutated
Unmutated
B
−log10(pvalue)
20
15
10
5
0
IGLV9-49
IGLV5-39
IGLV3-16
IGLV3-10
IGLV6-57
IGLV1-44
IGLV3-27
IGLV4-60
IGLV3-1
IGLV3-9
IGLV3-19
IGLV5-48
IGLV2-18
IGLV2-8
IGLV8-61
IGLV5-37
IGLJ1
IGLJ2-3ambi
IGLV7-46
IGLJ7
IGLV3-22
IGLV10-54
IGLV1-36
IGLV5-52
IGLV5-45
IGLV2-11
IGLJ6
IGLV3-21
IGLJ5
IGLV1-41
Repertoire
Mutated
Unmutated
C
IGK guQTL genes
14
14
4
IGL guQTL genes
7
19
4
Unmutated repertoire
Mutated repertoire
D
14 IGK guQTL genes
IGKJ
IGKV
Adjusted R²
0.8
0.6
0.4
0.2
Unmut
Mut
19 IGL guQTL genes
IGLJ
IGLV
Adjusted R²
0.4
0.3
0.2
0.1
Unmut
Mut

Figure S7

Mutated repertoires

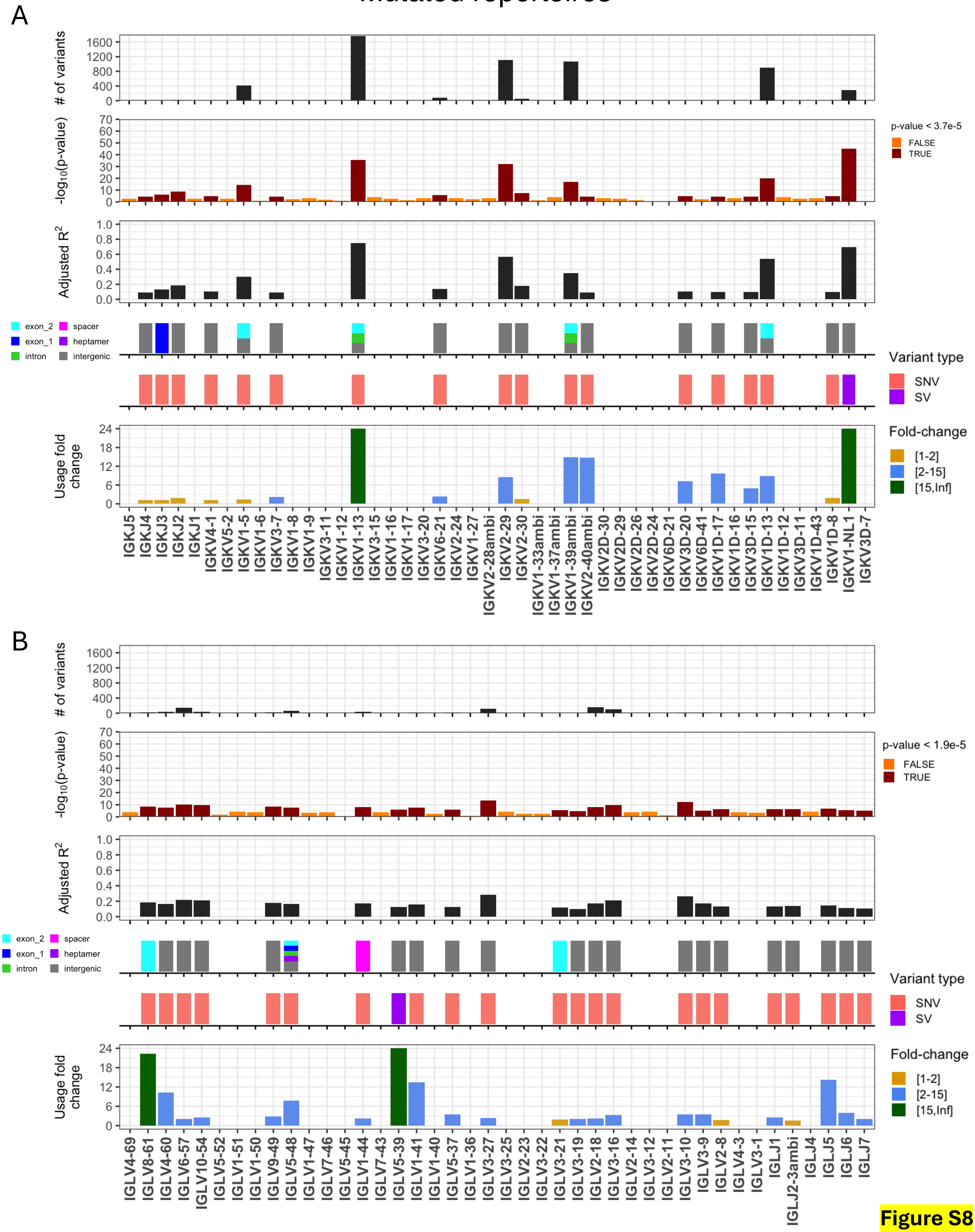

Figure S8

Figure S9

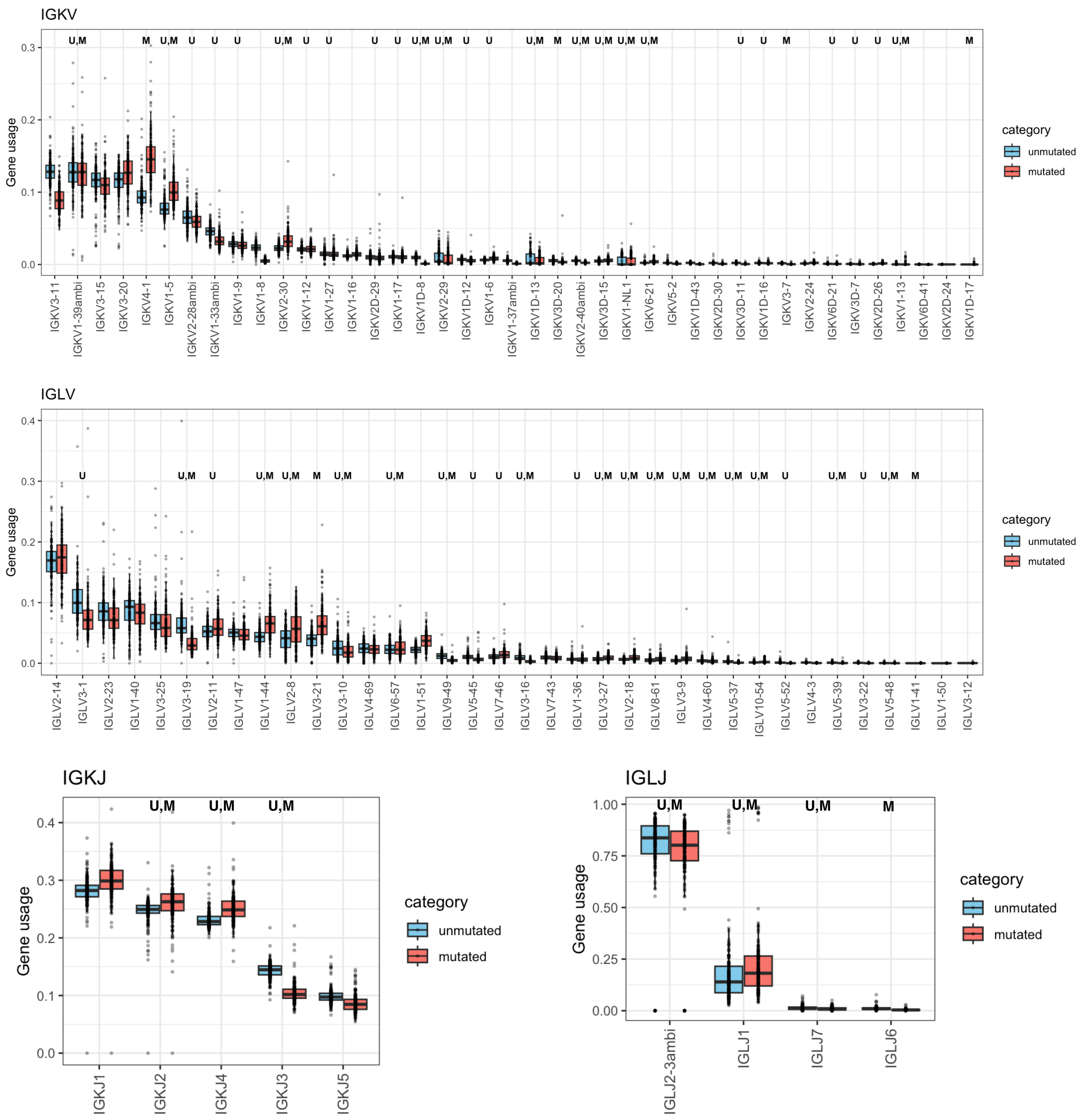
IGKV
Gene usage
IGKV3-11
IGKV1-39ambi
IGKV3-15
IGKV3-20
IGKV4-1
IGKV1-5
IGKV2-28ambi
IGKV1-33ambi
IGKV1-9
IGKV1-8
IGKV2-30
IGKV1-12
IGKV1-27
IGKV1-16
IGKV2D-29
IGKV1-17
IGKV1D-8
IGKV2-29
IGKV1D-12
IGKV1-6
IGKV1-37ambi
IGKV1D-13
IGKV3D-20
IGKV2-40ambi
IGKV3D-15
IGKV1-NL1
IGKV6-21
IGKV5-2
IGKV1D-43
IGKV2D-30
IGKV3D-11
IGKV1D-16
IGKV3-7
IGKV2-24
IGKV6D-21
IGKV3D-7
IGKV2D-26
IGKV1-13
IGKV6D-41
IGKV2D-24
IGKV1D-17
category
unmutated
mutated
IGLV
Gene usage
IGLV2-14
IGLV3-1
IGLV2-23
IGLV1-40
IGLV3-25
IGLV3-19
IGLV2-11
IGLV1-47
IGLV1-44
IGLV2-8
IGLV3-21
IGLV3-10
IGLV4-69
IGLV6-57
IGLV1-51
IGLV9-49
IGLV5-45
IGLV7-46
IGLV3-16
IGLV7-43
IGLV1-36
IGLV3-27
IGLV2-18
IGLV8-61
IGLV3-9
IGLV4-60
IGLV5-37
IGLV10-54
IGLV5-52
IGLV4-3
IGLV5-39
IGLV3-22
IGLV5-48
IGLV1-41
IGLV1-50
IGLV3-12
category
unmutated
mutated
IGKJ
Gene usage
IGKJ1
IGKJ2
IGKJ4
IGKJ3
IGKJ5
category
unmutated
mutated
IGLJ
Gene usage
IGLJ2-3ambi
IGLJ1
IGLJ7
IGLJ6
category
unmutated
mutated

Figure S10

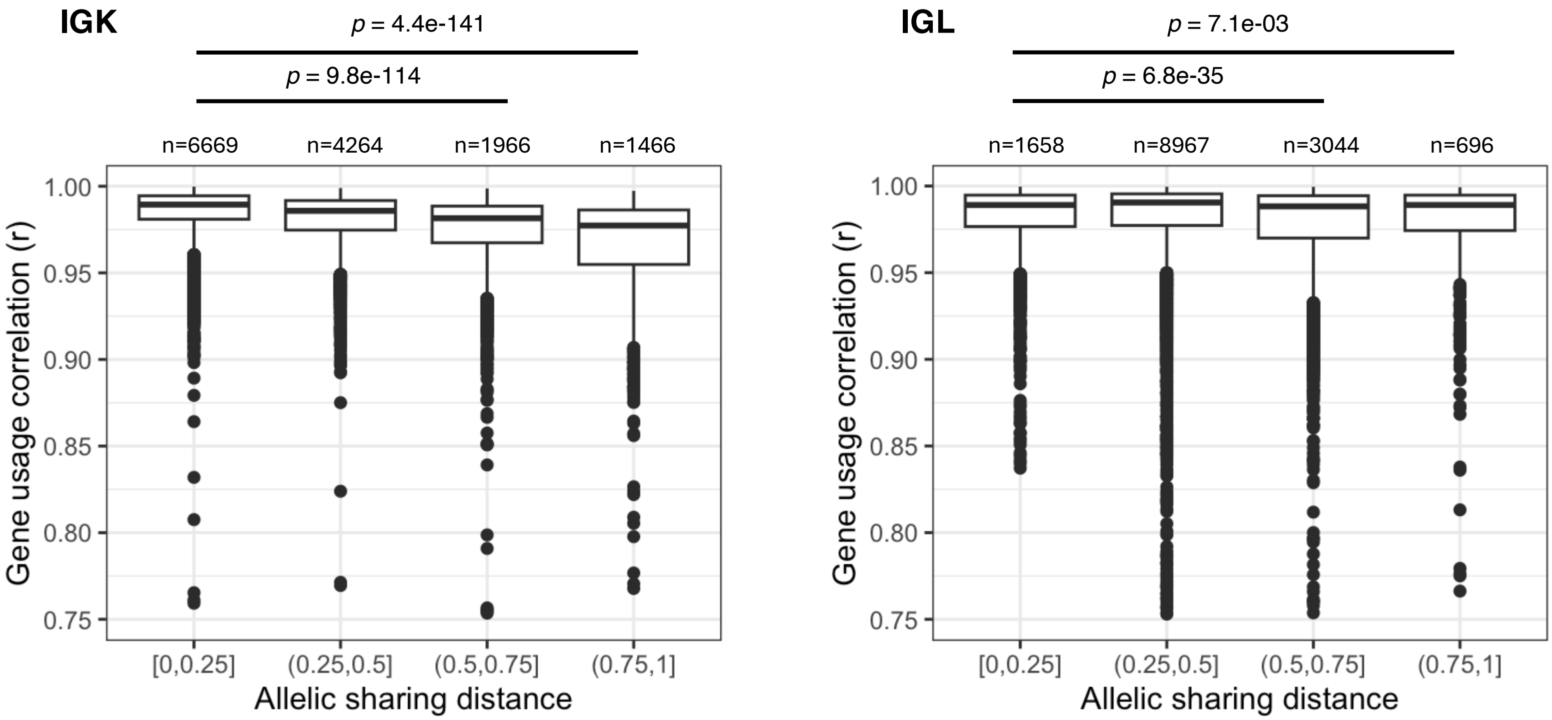
IGK
p = 4.4e-141
p = 9.8e-114
n=6669
n=4264
n=1966
n=1466
Gene usage correlation (r)
1.00
0.95
0.90
0.85
0.80
0.75
[0,0.25]
(0.25,0.5]
(0.5,0.75]
(0.75,1]
Allelic sharing distance
IGL
p = 7.1e-03
p = 6.8e-35
n=1658
n=8967
n=3044
n=696
Gene usage correlation (r)
1.00
0.95
0.90
0.85
0.80
0.75
[0,0.25]
(0.25,0.5]
(0.5,0.75]
(0.75,1]
Allelic sharing distance

Figure S11

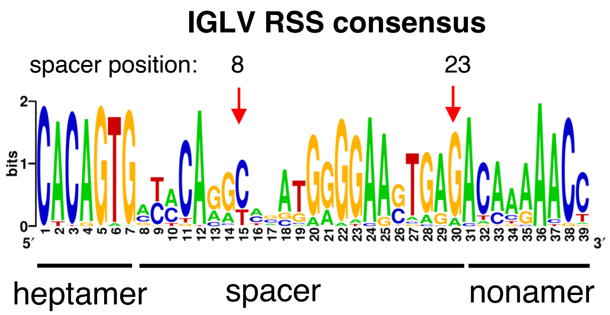

IGLV RSS consensus
spacer position: 8
23
bits
2
1
0
5′
3′
heptamer
spacer
nonamer

Figure S12

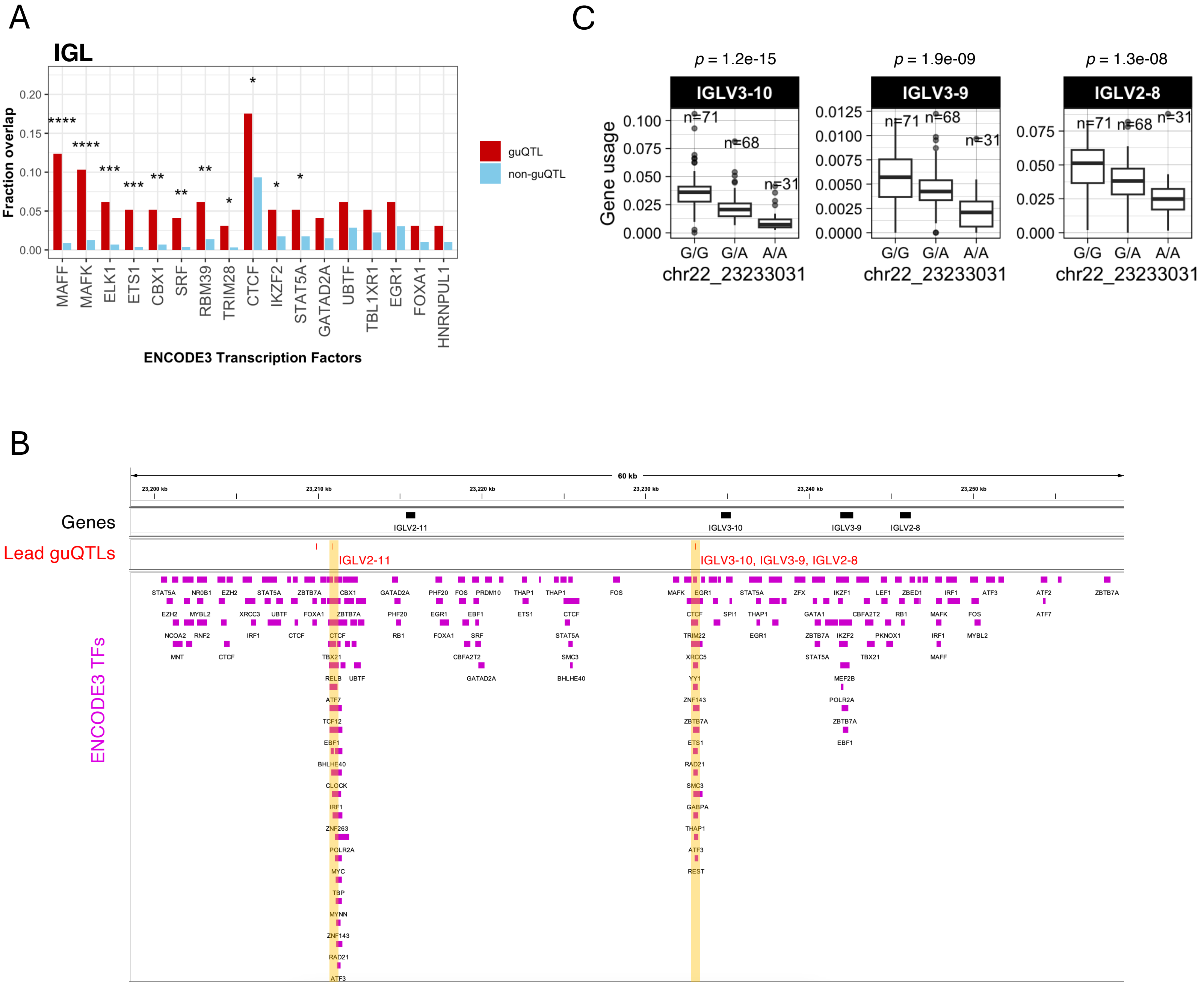
A
IGL
Fraction overlap
0.20
0.15
0.10
0.05
0.00
****
****
***
***
**
**
**
*
*
*
*
guQTL
non-guQTL
MAFF
MAFK
ELK1
ETS1
CBX1
SRF
RBM39
TRIM28
CTCF
IKZF2
STAT5A
GATAD2A
UBTF
TBL1XR1
EGR1
FOXA1
HNRNPUL1
ENCODE3 Transcription Factors
C
p = 1.2e-15
p = 1.9e-09
p = 1.3e-08
IGLV3-10
IGLV3-9
IGLV2-8
Gene usage
n=71
n=68
n=31
G/G
G/A
A/A
chr22_23233031
B
60 kb
Genes
IGLV2-11
IGLV3-10
IGLV3-9
IGLV2-8
Lead guQTLs
IGLV2-11
IGLV3-10, IGLV3-9, IGLV2-8
ENCODE3 TFs

Figure S13

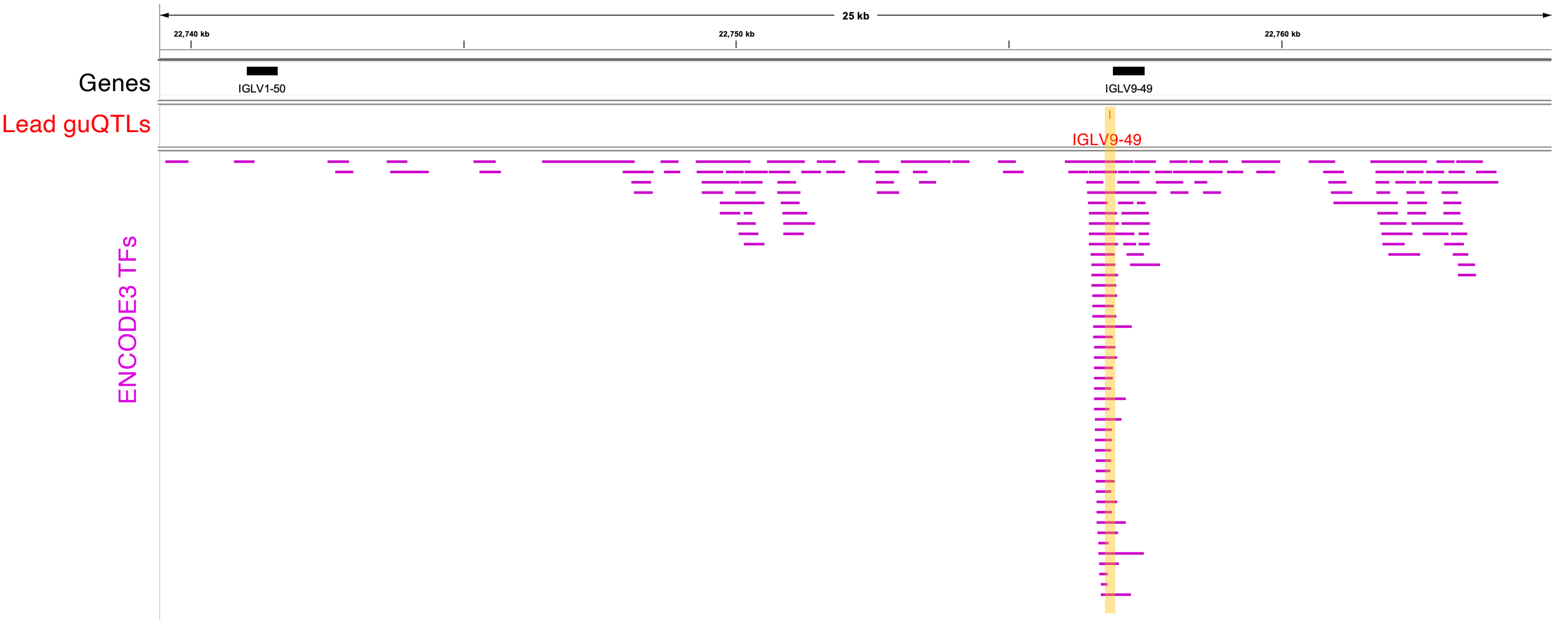

25 kb
22,740 kb
22,750 kb
22,760 kb
Genes
IGLV1-50
IGLV9-49
Lead guQTLs
IGLV9-49
ENCODE3 TFs

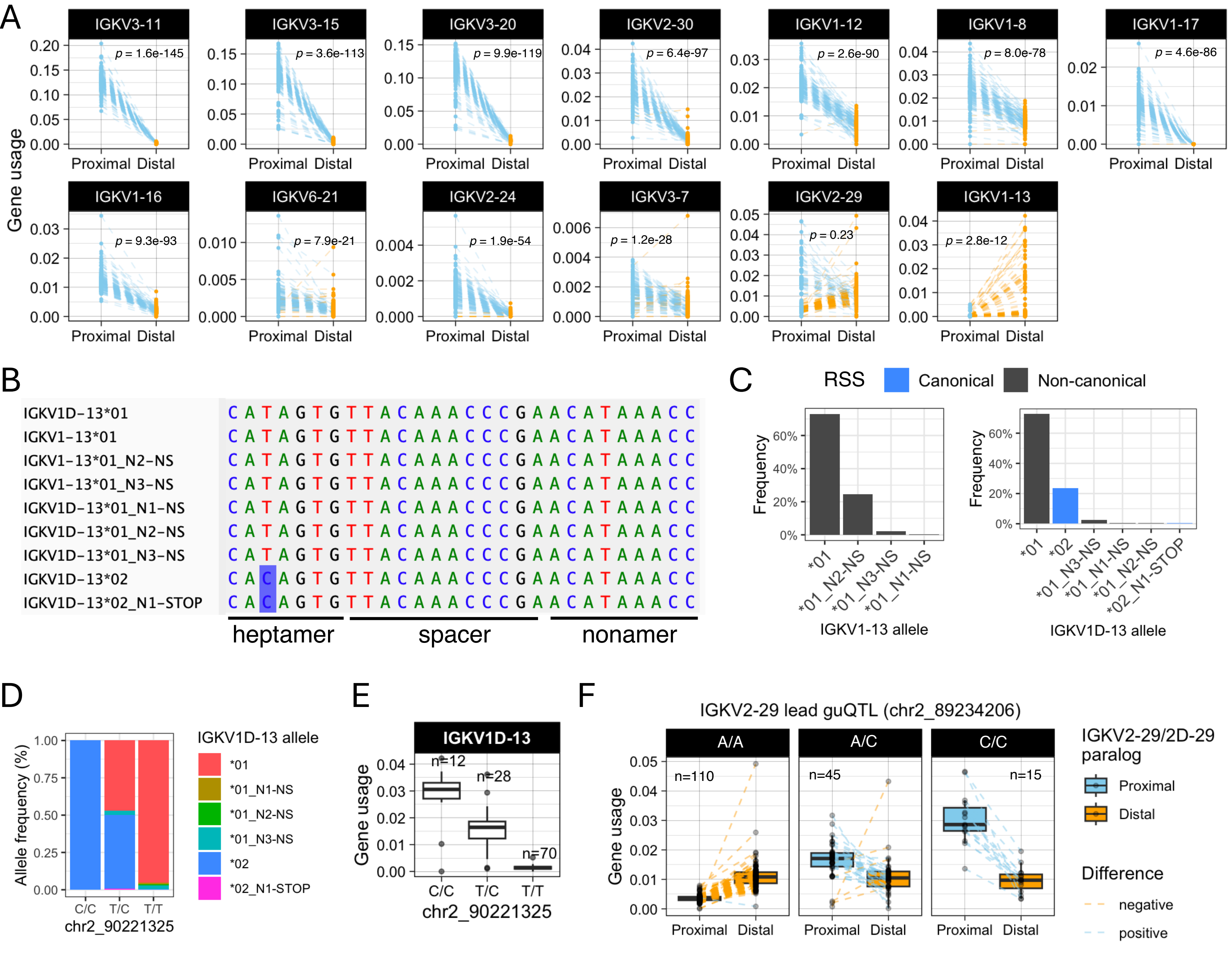
A
IGKV3-11
p = 1.6e-145
IGKV3-15
p = 3.6e-113
IGKV3-20
p = 9.9e-119
IGKV2-30
p = 6.4e-97
IGKV1-12
p = 2.6e-90
IGKV1-8
p = 8.0e-78
IGKV1-17
p = 4.6e-86
IGKV1-16
p = 9.3e-93
IGKV6-21
p = 7.9e-21
IGKV2-24
p = 1.9e-54
IGKV3-7
p = 1.2e-28
IGKV2-29
p = 0.23
IGKV1-13
p = 2.8e-12
Gene usage
Proximal Distal
B
IGKV1D-13*01
IGKV1-13*01
IGKV1-13*01_N2-NS
IGKV1-13*01_N3-NS
IGKV1D-13*01_N1-NS
IGKV1D-13*01_N2-NS
IGKV1D-13*01_N3-NS
IGKV1D-13*02
IGKV1D-13*02_N1-STOP
CATAGTGTTACAAACCCGAACATAAACC
CACAGTGTTACAAACCCGAACATAAACC
heptamer
spacer
nonamer
C
RSS
Canonical
Non-canonical
Frequency
IGKV1-13 allele
*01
*01_N2-NS
*01_N3-NS
*01_N1-NS
IGKV1D-13 allele
*01
*02
*01_N3-NS
*01_N1-NS
*01_N2-NS
*02_N1-STOP
D
Allele frequency (%)
chr2_90221325
C/C
T/C
T/T
IGKV1D-13 allele
*01
*01_N1-NS
*01_N2-NS
*01_N3-NS
*02
*02_N1-STOP
E
IGKV1D-13
Gene usage
n=12
n=28
n=70
C/C
T/C
T/T
chr2_90221325
F
IGKV2-29 lead guQTL (chr2_89234206)
A/A
A/C
C/C
n=110
n=45
n=15
Gene usage
Proximal Distal
IGKV2-29/2D-29 paralog
Proximal
Distal
Difference
negative
positive

Figure S14

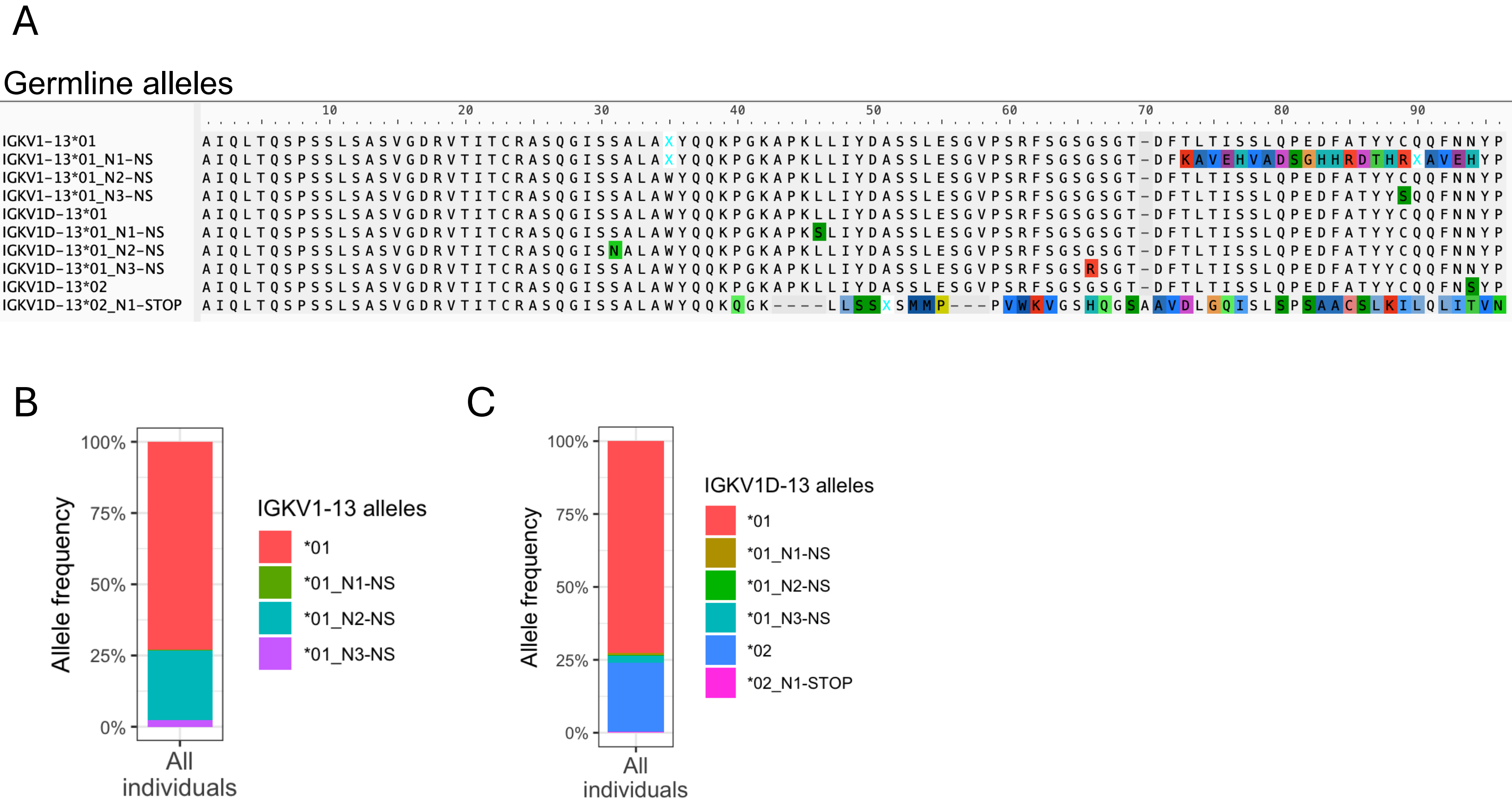
A
Germline alleles
10
20
30
40
50
60
70
80
90
IGKV1-13*01 AIQLTQSPSSLSASVGDRVTITCRASQGISSALAXYQQKPGKAPKLLIYDASSLESGVPSRFSGSGSGT-DFTLTISSLQPEDFATYYCQQFNNYP
IGKV1-13*01_N1-NS AIQLTQSPSSLSASVGDRVTITCRASQGISSALAXYQQKPGKAPKLLIYDASSLESGVPSRFSGSGSGT-DFKAVEHVADSGHHRDTHRXAVEHYP
IGKV1-13*01_N2-NS AIQLTQSPSSLSASVGDRVTITCRASQGISSALAWYQQKPGKAPKLLIYDASSLESGVPSRFSGSGSGT-DFTLTISSLQPEDFATYYCQQFNNYP
IGKV1-13*01_N3-NS AIQLTQSPSSLSASVGDRVTITCRASQGISSALAWYQQKPGKAPKLLIYDASSLESGVPSRFSGSGSGT-DFTLTISSLQPEDFATYYSQQFNNYP
IGKV1D-13*01 AIQLTQSPSSLSASVGDRVTITCRASQGISSALAWYQQKPGKAPKLLIYDASSLESGVPSRFSGSGSGT-DFTLTISSLQPEDFATYYCQQFNNYP
IGKV1D-13*01_N1-NS AIQLTQSPSSLSASVGDRVTITCRASQGISSALAWYQQKPGKAPKSLIYDASSLESGVPSRFSGSGSGT-DFTLTISSLQPEDFATYYCQQFNNYP
IGKV1D-13*01_N2-NS AIQLTQSPSSLSASVGDRVTITCRASQGISNALAWYQQKPGKAPKLLIYDASSLESGVPSRFSGSGSGT-DFTLTISSLQPEDFATYYCQQFNNYP
IGKV1D-13*01_N3-NS AIQLTQSPSSLSASVGDRVTITCRASQGISSALAWYQQKPGKAPKLLIYDASSLESGVPSRFSGSRSGT-DFTLTISSLQPEDFATYYCQQFNNYP
IGKV1D-13*02 AIQLTQSPSSLSASVGDRVTITCRASQGISSALAWYQQKPGKAPKLLIYDASSLESGVPSRFSGSGSGT-DFTLTISSLQPEDFATYYCQQFNSYP
IGKV1D-13*02_N1-STOP AIQLTQSPSSLSASVGDRVTITCRASQGISSALAWYQQKQGK----LLSSXSMMP---PVWKVGSHQGSAAVDLGQISLSPSAACSLKILQLITVN
B
100%
75%
50%
25%
0%
Allele frequency
All individuals
IGKV1-13 alleles
*01
*01_N1-NS
*01_N2-NS
*01_N3-NS
C
100%
75%
50%
25%
0%
Allele frequency
All individuals
IGKV1D-13 alleles
*01
*01_N1-NS
*01_N2-NS
*01_N3-NS
*02
*02_N1-STOP

Figure S16

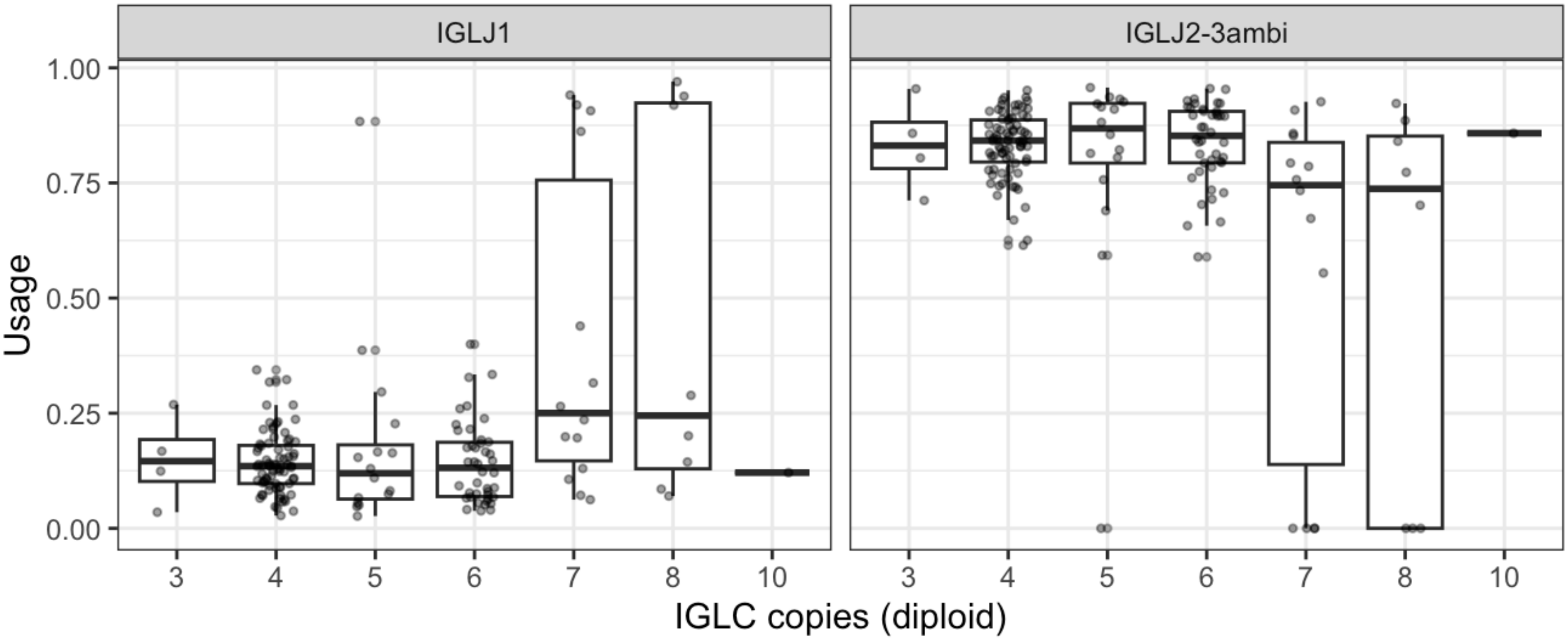

P = 5.17E-06 (linear regression)
P = 1.38E-08 (ANOVA)

P = 1.29E-05 (linear regression)
P = 4.46E-08 (ANOVA)

Figure S17

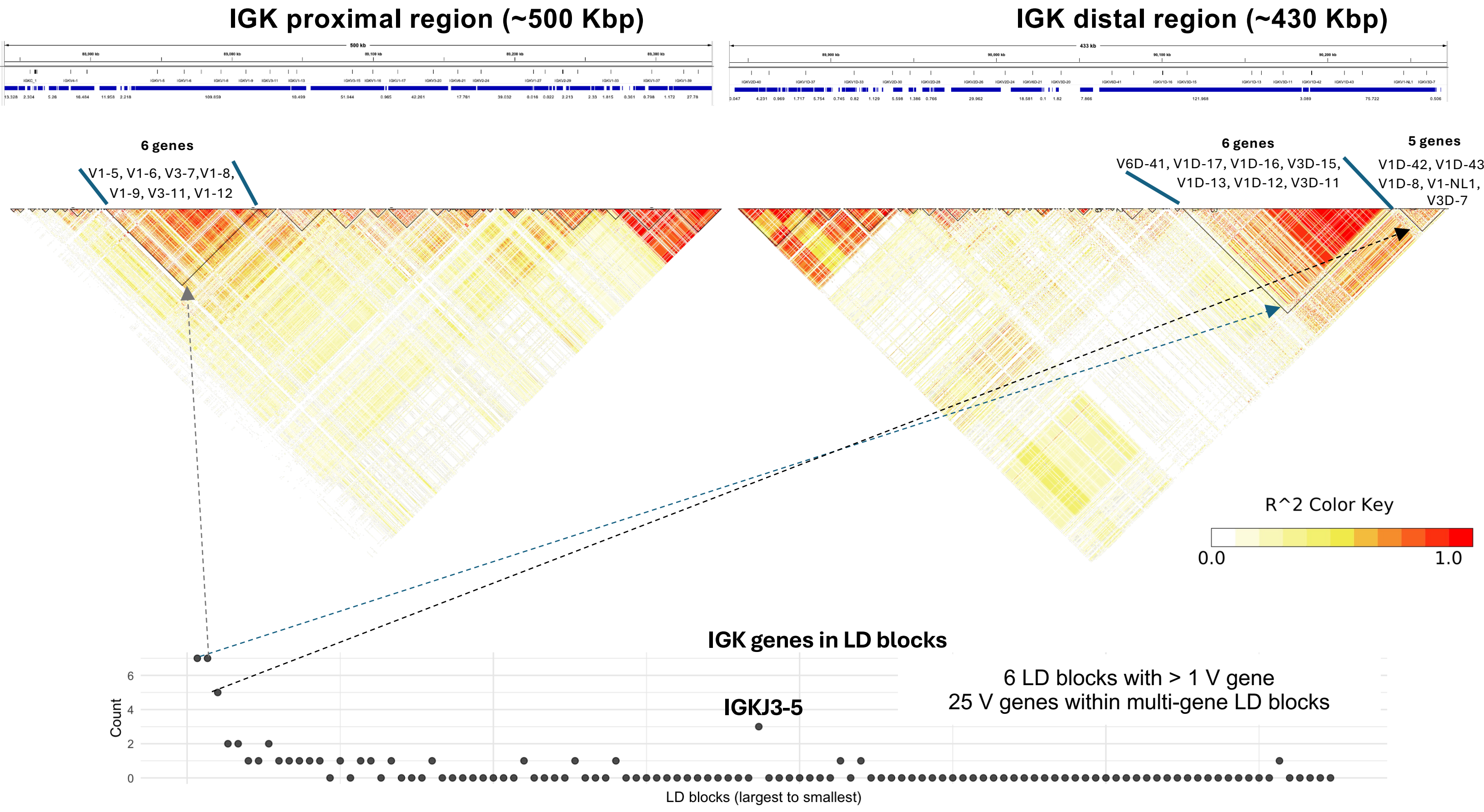
IGK proximal region (~500 Kbp)
IGK distal region (~430 Kbp)
6 genes
V1-5, V1-6, V3-7,V1-8,
V1-9, V3-11, V1-12
6 genes
V6D-41, V1D-17, V1D-16, V3D-15,
V1D-13, V1D-12, V3D-11
5 genes
V1D-42, V1D-43
V1D-8, V1-NL1,
V3D-7
R^2 Color Key
0.0
1.0
IGK genes in LD blocks
6 LD blocks with > 1 V gene
25 V genes within multi-gene LD blocks
IGKJ3-5
Count
LD blocks (largest to smallest)

Figure S18

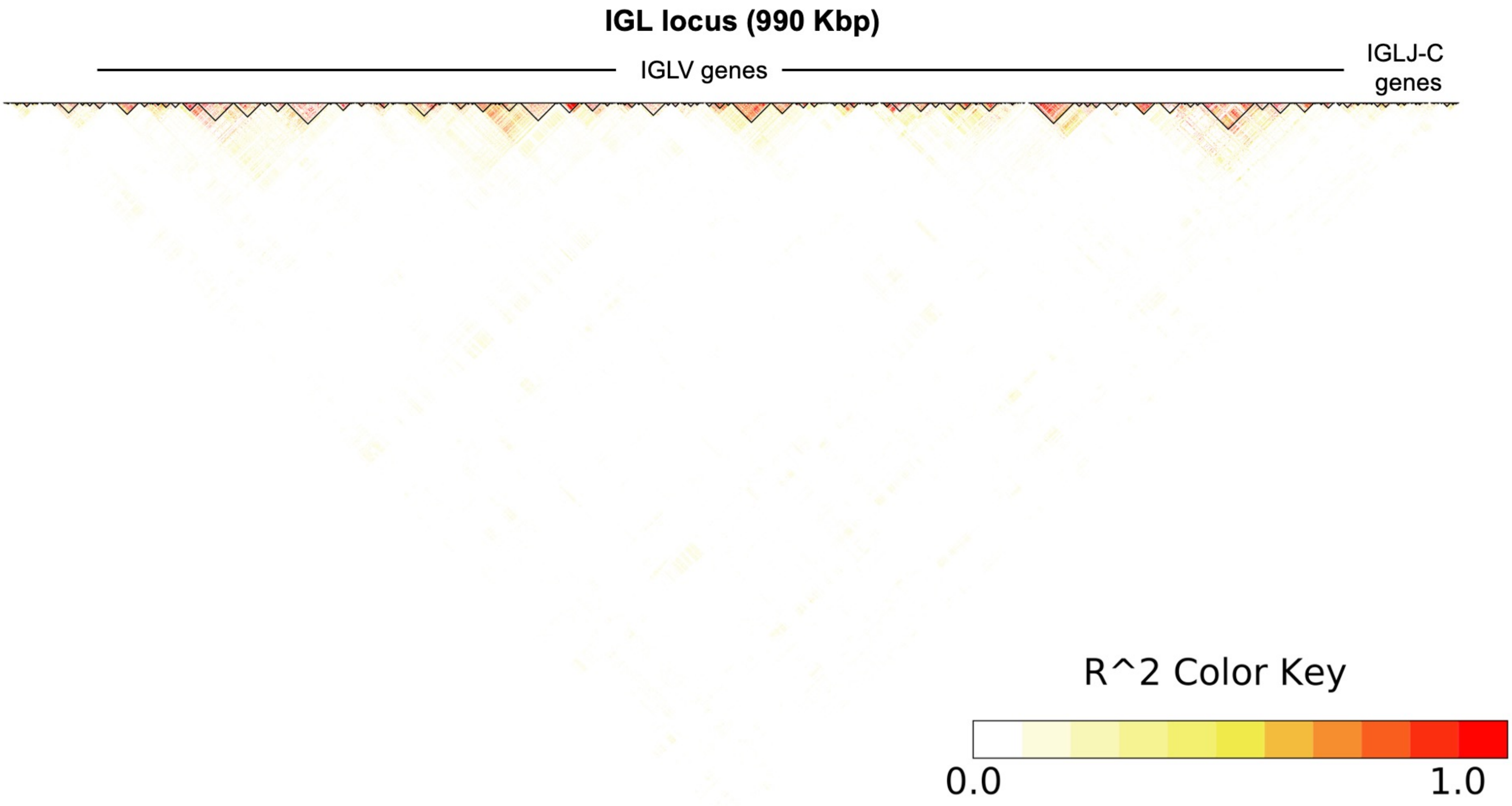

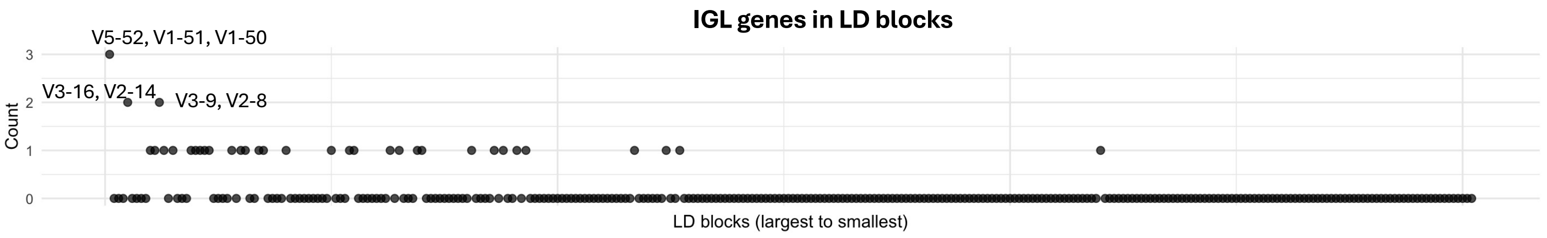

3 LD blocks with > 1 V gene
7 V genes within multi-gene LD blocks

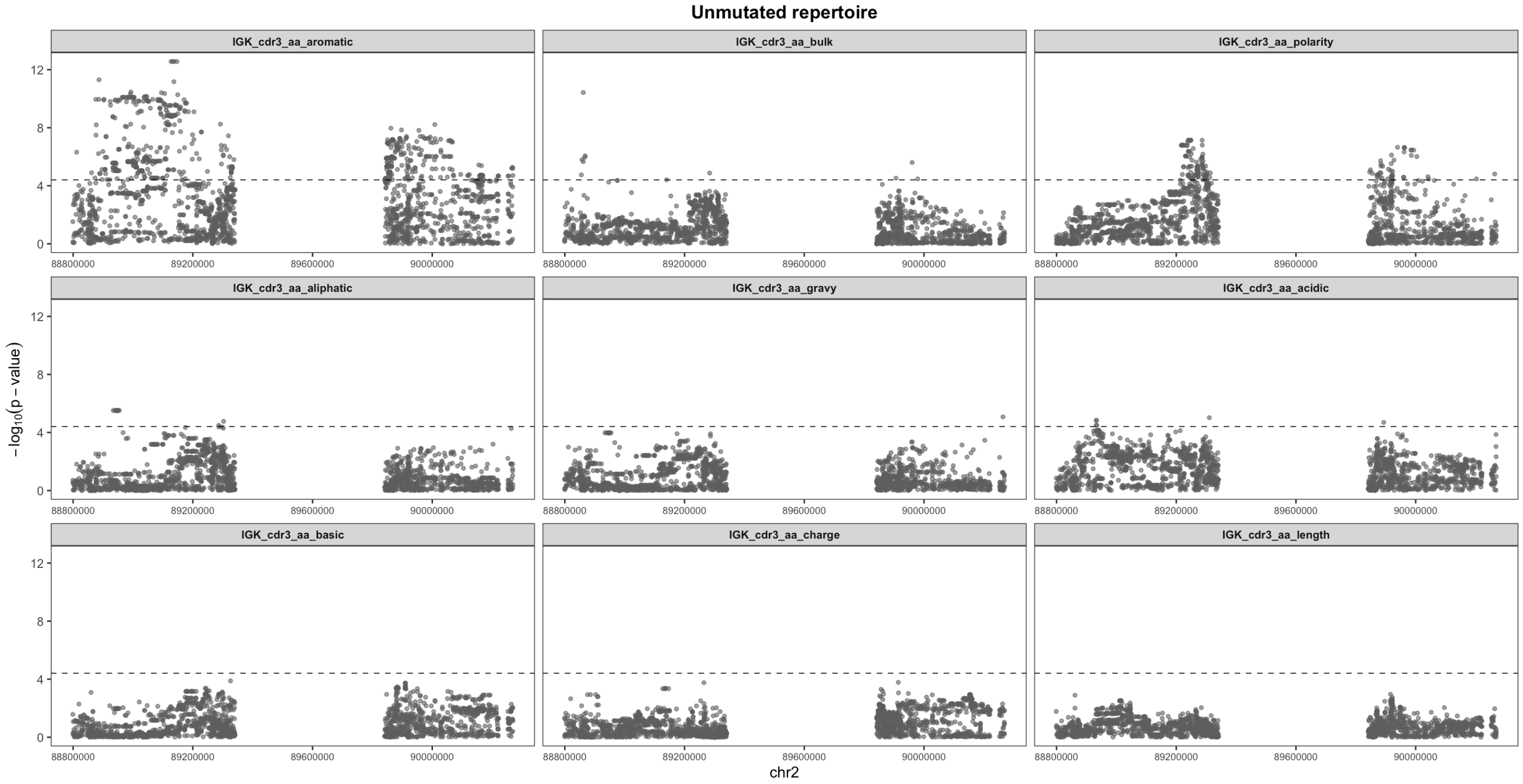
Unmutated repertoire
IGK_cdr3_aa_aromatic
IGK_cdr3_aa_bulk
IGK_cdr3_aa_polarity
IGK_cdr3_aa_aliphatic
IGK_cdr3_aa_gravy
IGK_cdr3_aa_acidic
IGK_cdr3_aa_basic
IGK_cdr3_aa_charge
IGK_cdr3_aa_length
−log10(p − value)
chr2
88800000
89200000
89600000
90000000
0
4
8
12

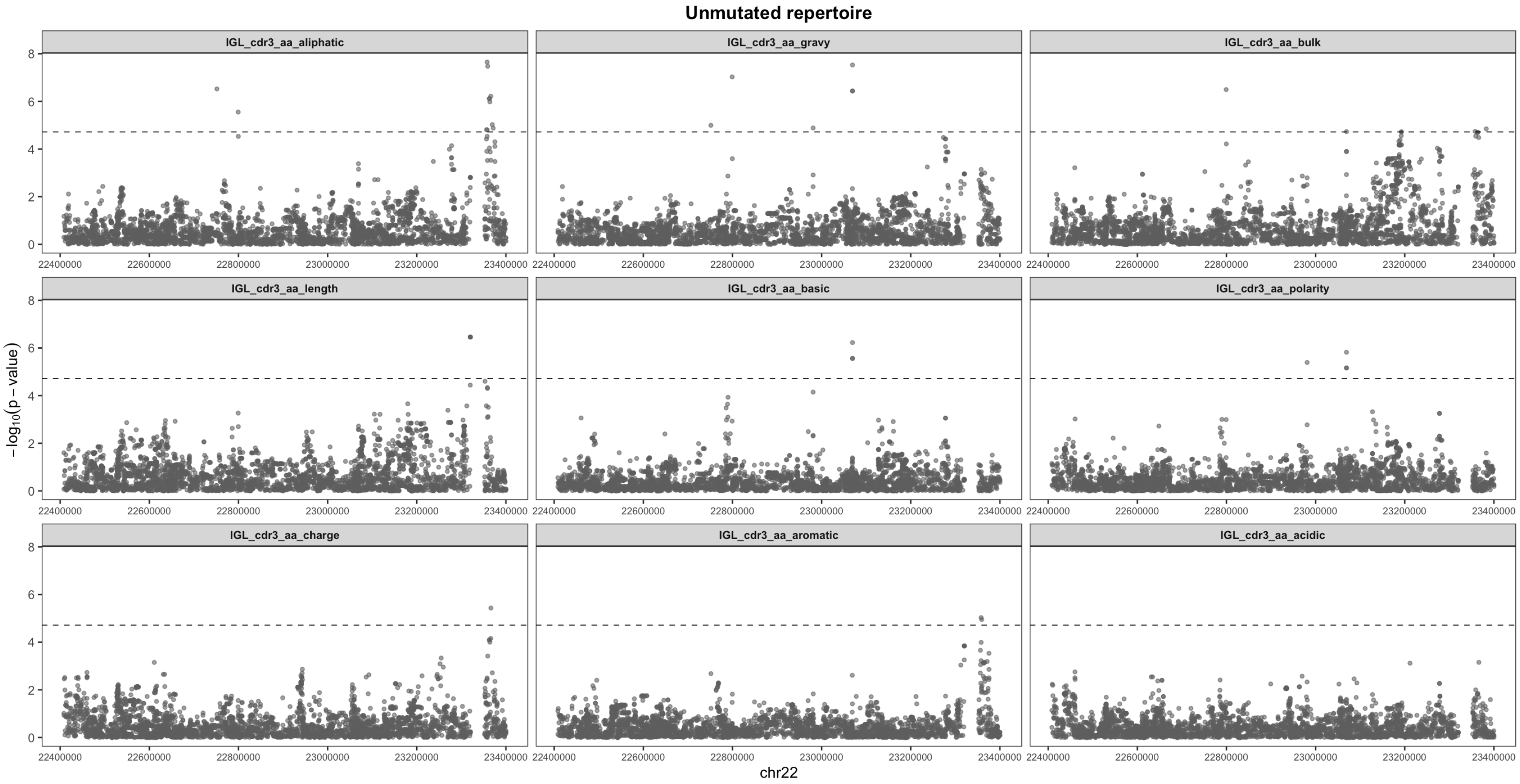
Unmutated repertoire
IGL_cdr3_aa_aliphatic
IGL_cdr3_aa_gravy
IGL_cdr3_aa_bulk
IGL_cdr3_aa_length
IGL_cdr3_aa_basic
IGL_cdr3_aa_polarity
IGL_cdr3_aa_charge
IGL_cdr3_aa_aromatic
IGL_cdr3_aa_acidic
−log10(p − value)
chr22
22400000
22600000
22800000
23000000
23200000
23400000
0
2
4
6
8

Figure S19

Figure S20

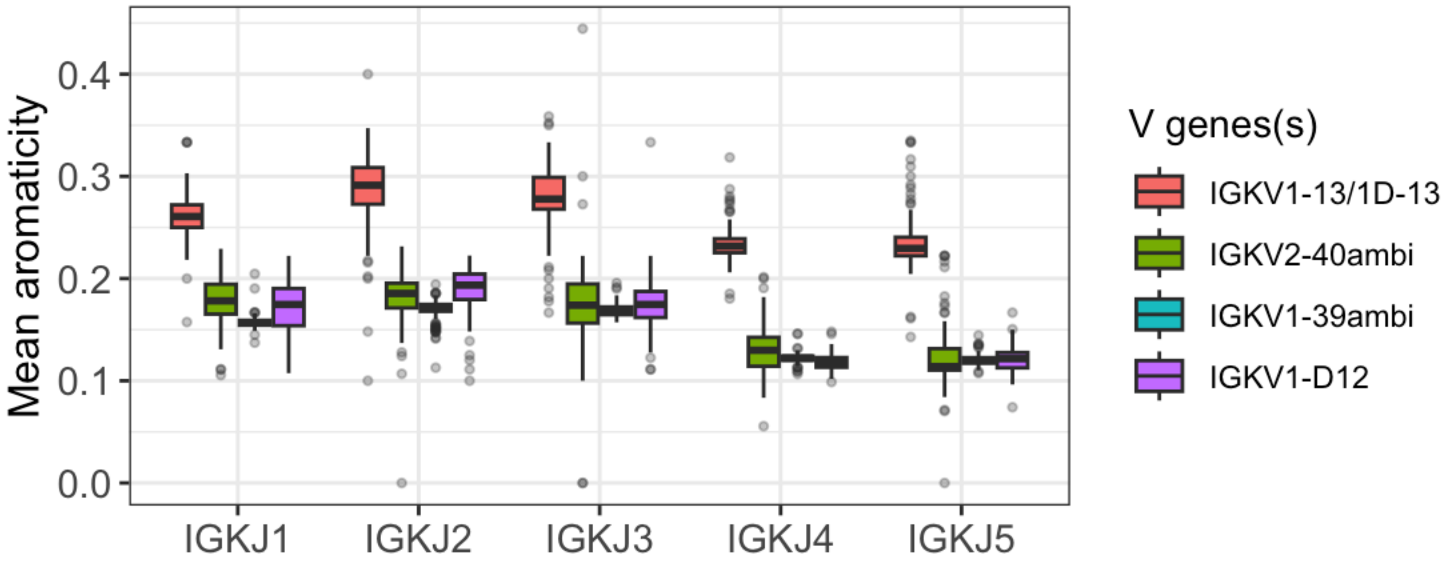
Mean aromaticity
0.0
0.1
0.2
0.3
0.4
IGKJ1
IGKJ2
IGKJ3
IGKJ4
IGKJ5
V genes(s)
IGKV1-13/1D-13
IGKV2-40ambi
IGKV1-39ambi
IGKV1-D12

Figure S21

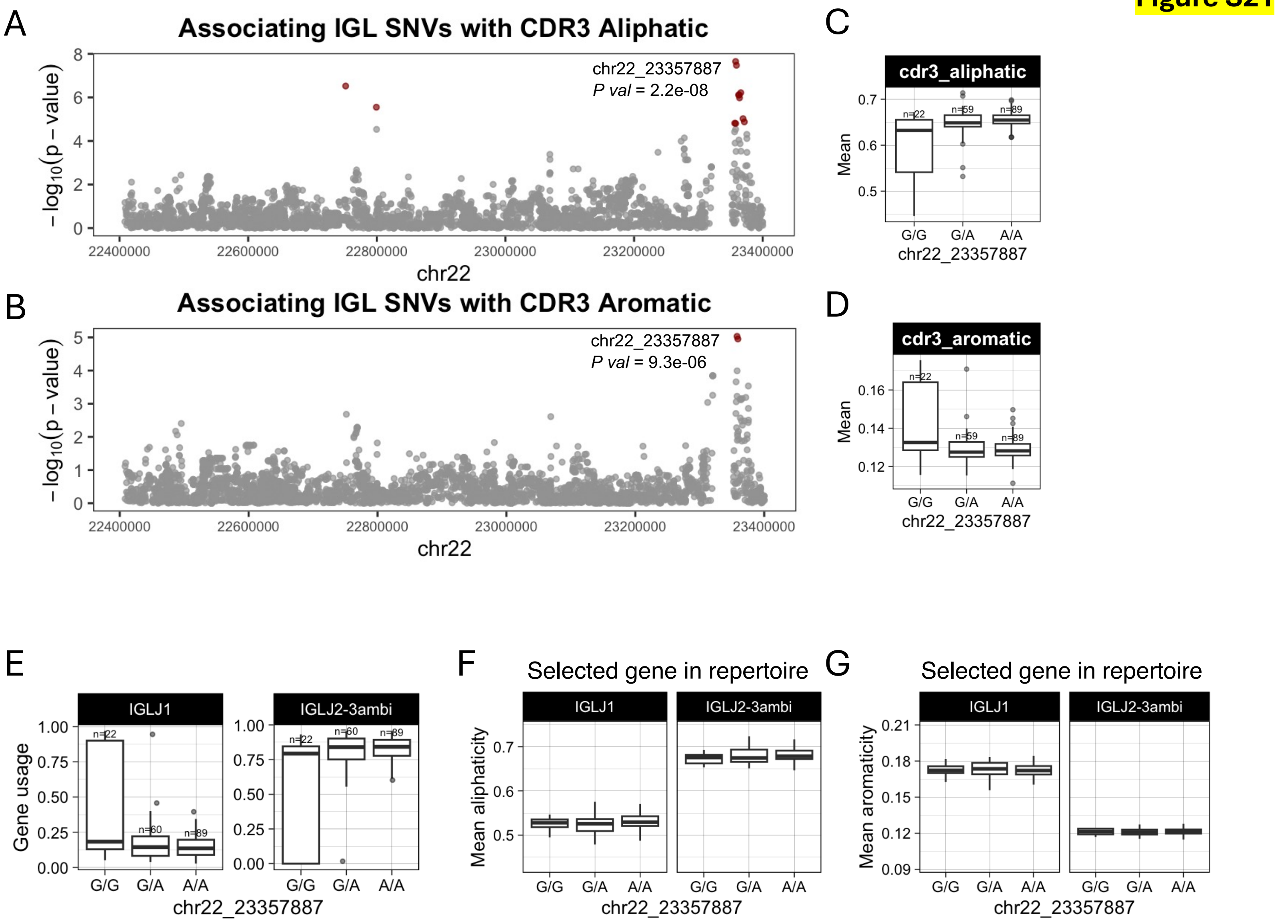
A
Associating IGL SNVs with CDR3 Aliphatic
chr22_23357887
P val = 2.2e-08
− log10(p − value)
chr22
B
Associating IGL SNVs with CDR3 Aromatic
chr22_23357887
P val = 9.3e-06
− log10(p − value)
chr22
C
cdr3_aliphatic
Mean
G/G
G/A
A/A
chr22_23357887
D
cdr3_aromatic
Mean
G/G
G/A
A/A
chr22_23357887
E
IGLJ1
IGLJ2-3ambi
Gene usage
chr22_23357887
F
Selected gene in repertoire
IGLJ1
IGLJ2-3ambi
Mean aliphaticity
chr22_23357887
G
Selected gene in repertoire
IGLJ1
IGLJ2-3ambi
Mean aromaticity
chr22_23357887
