# Supplementary material for: Germline polymorphism in the immunoglobulin kappa and lambda loci explain variation in the expressed light chain antibody repertoire
Source: bioRxiv. 2025 Jun 1:2025.05.28.656470. Preprint. [Version 1] doi: 10.1101/2025.05.28.656470 (PMC12154815; doi:10.1101/2025.05.28.656470)
Supplement: Supplementary file 2 [file media-2.docx]

**Identification of SVs, SNVs, and gene alleles in IGK and IGL using long-read sequencing**

To genotype variants in IGK and IGL, we used probe-based targeted capture long-read single molecule real-time (SMRT) sequencing [(](https://paperpile.com/c/fJySTi/R4hl)[*1*](https://paperpile.com/c/fJySTi/R4hl)[)](https://paperpile.com/c/fJySTi/R4hl) of the IGK proximal and distal regions [(](https://paperpile.com/c/fJySTi/7XJkC)[*2*](https://paperpile.com/c/fJySTi/7XJkC)[)](https://paperpile.com/c/fJySTi/7XJkC), and the IGL locus [(](https://paperpile.com/c/fJySTi/RgYut)[*3*](https://paperpile.com/c/fJySTi/RgYut)[)](https://paperpile.com/c/fJySTi/RgYut). IGK was sequenced to a coverage of 65.9X on average, with a mean of 1,858,094 diploid base pairs assembled per individual and mean assembly accuracy of 99.96%. IGL was sequenced to a coverage of 41.3X on average, with a mean of 1,903,810 diploid base pairs assembled per individual and mean assembly accuracy of 99.93% (**Supplementary Table S1, Supplementary Figure 1**).

One of the primary objectives of this study was to develop a high-confidence set of genetic variants in IGK and IGL to enable downstream genetic association analysis. We previously described common SVs in IGK [(](https://paperpile.com/c/fJySTi/7XJkC)[*2*](https://paperpile.com/c/fJySTi/7XJkC)[)](https://paperpile.com/c/fJySTi/7XJkC) and IGL [(](https://paperpile.com/c/fJySTi/RgYut)[*3*](https://paperpile.com/c/fJySTi/RgYut)[)](https://paperpile.com/c/fJySTi/RgYut), including polymorphisms associated with gene conversion in IGK, a large inversion in IGK, a deletion involving IGKV1-NL1, a deletion involving IGLV5-39, and a deletion involving IGLV3-16, IGLV2-18, IGLV3-19, IGLV3-21, IGLV3-22, and IGLV2-23 (termed IGLV2-18) (**Supplementary Figure 2A-B**). 

In addition to these SVs, we identified a haplotype wherein the entire IGKV distal region is deleted, which was previously reported [(](https://paperpile.com/c/fJySTi/Z3QuC+O69c9)[*4*](https://paperpile.com/c/fJySTi/Z3QuC+O69c9)[, ](https://paperpile.com/c/fJySTi/Z3QuC+O69c9)[*5*](https://paperpile.com/c/fJySTi/Z3QuC+O69c9)[)](https://paperpile.com/c/fJySTi/Z3QuC+O69c9), removing 23 functional IGKV genes. Nine individuals with this deletion haplotype exhibited ~2-3-fold higher sequencing coverage over the proximal relative to distal region (**Supplementary Figure 4**), suggestive of hemizygosity. An additional individual lacked both HiFi reads and assemblies mapped to the IGK distal region, despite having 87X coverage and diploid assemblies for the proximal region (**Supplementary Figure 4**), indicating homozygous absence of the IGKV distal region in this sample. SV allele frequencies for samples in this cohort are summarized in **Supplemental Table S2**. 

SNVs within segmental duplications are difficult to characterize. We previously demonstrated that SNV callsets from haplotype-resolved IGK [(](https://paperpile.com/c/fJySTi/7XJkC)[*2*](https://paperpile.com/c/fJySTi/7XJkC)[)](https://paperpile.com/c/fJySTi/7XJkC) and IGL [(](https://paperpile.com/c/fJySTi/RgYut)[*3*](https://paperpile.com/c/fJySTi/RgYut)[)](https://paperpile.com/c/fJySTi/RgYut) assemblies derived from long-read SMRT sequencing outperform short-read derived variant calls in phase 3 [(](https://paperpile.com/c/fJySTi/oZBB1)[*6*](https://paperpile.com/c/fJySTi/oZBB1)[)](https://paperpile.com/c/fJySTi/oZBB1) of the 1KGP. We identified variants for each individual and generated a callset of common SNVs, defined as those with a minor allele frequency (MAF) ≥ 0.05, totaling 2,792 and 5,198 common SNVs in IGK and IGL, respectively (**Supplementary Figure 2C**). Comparison of these SNVs with those in the dbSNP “common” (MAF ≥ 0.01) catalog revealed substantial differences; 59.3% and 38.8% of common SNVs in IGK and IGL, respectively, were absent from this dbSNP catalog (**Supplementary Figure 2D-E**). These data indicate that dbSNP lacks accurate genotype information for about half of the common variants in IGK and about a third of the common variants in IGL. 

While the majority of common SNVs were intergenic (IGK, 95%; IGL, 97%), SNVs were identified within features of V and J genes, including coding exons, V gene introns, and RSS heptamers, spacers, and nonamers (**Supplementary Figure 5**). 34 of 47 IGKV genes (72%) and 29 of 39 IGLV genes (74%) harbored at least one common SNV within a gene feature.  

	Analysis of AIRR-seq data critically relies on assignment of AIRR-seq reads to specific IG gene alleles, which are typically identified from a germline allele database. For a given individual, accurate AIRR-seq analysis requires inclusion of all of the individual’s alleles in the germline database to permit accurate assignment of reads to gene alleles; these assignments are used for analyzing a variety of Ab repertoire features, including gene usage and somatic hypermutation. We previously demonstrated that there is significant variation among the IGK [(](https://paperpile.com/c/fJySTi/7XJkC)[*2*](https://paperpile.com/c/fJySTi/7XJkC)[)](https://paperpile.com/c/fJySTi/7XJkC) and IGL [(](https://paperpile.com/c/fJySTi/RgYut)[*3*](https://paperpile.com/c/fJySTi/RgYut)[)](https://paperpile.com/c/fJySTi/RgYut) gene alleles in the human population, and many of these alleles are not documented in the commonly used germline alleles database called the ImMunoGeneTics Information System (IMGT; imgt.org). With haplotype-resolved IG assemblies in-hand, one can use a personalized germline allele database for AIRR-seq analysis of the individual.  

	To annotate germline IGK and IGL gene alleles in this cohort, we used haplotype-resolved assemblies and identified both documented and undocumented (novel) alleles, defined as alleles absent from IMGT (https://www.imgt.org). In total, we identified 160 and 145 high-confidence novel IGKV and IGLV alleles, respectively, defined as alleles with exact matches to ≥10 HiFi reads that mapped to the position of the allele sequence in the assembly (**Supplementary Figure 2F-I**; **Supplementary Table S3**, **Supplementary Table S4**). Among all IGKV and IGLV alleles, 67.4% and 62.8% were not documented in IMGT (**Supplementary Figure 6**). Among the novel alleles, 81 IGKV and 70 IGLV alleles were identified in more than 1 individual (**Supplementary Figure 2H-I**). The majority of novel alleles resulted in non-synonymous substitutions (as compared to the closest-matching allele in IMGT), with 7 IGKV and 7 IGLV novel alleles encoding premature STOP codons (**Supplementary Figure 2H-I**). We noted that 44 novel IGKV alleles were also identified in our previous survey of IGKV alleles using samples collected as part of the 1KGP [(](https://paperpile.com/c/fJySTi/7XJkC)[*2*](https://paperpile.com/c/fJySTi/7XJkC)[)](https://paperpile.com/c/fJySTi/7XJkC). To access and explore curated genetic resources from this dataset further see Peres et al. (in prep). 

**IGK and IGL repertoire-wide gene usage profiles are more highly correlated in individuals carrying shared genotypes**

	Monozygotic twin studies have shown that gene usage frequencies in genetically identical individuals correlated to a greater degree than in unrelated individuals [(](https://paperpile.com/c/fJySTi/fAZZE+7OQpo+AQIqn)[*7*](https://paperpile.com/c/fJySTi/fAZZE+7OQpo+AQIqn)[–](https://paperpile.com/c/fJySTi/fAZZE+7OQpo+AQIqn)[*9*](https://paperpile.com/c/fJySTi/fAZZE+7OQpo+AQIqn)[)](https://paperpile.com/c/fJySTi/fAZZE+7OQpo+AQIqn). For IGH, we extended this observation at the population level and demonstrated that repertoire-wide gene usage profiles are more highly correlated in individuals carrying shared genotypes at guQTL SNVs [(](https://paperpile.com/c/fJySTi/r6wFt)[*10*](https://paperpile.com/c/fJySTi/r6wFt)[)](https://paperpile.com/c/fJySTi/r6wFt). To assess this in IGK and IGL, we used the same approach by estimating allele sharing distance (ASD) [(](https://paperpile.com/c/fJySTi/ZEXRx+dEgzw)[*11*](https://paperpile.com/c/fJySTi/ZEXRx+dEgzw)[, ](https://paperpile.com/c/fJySTi/ZEXRx+dEgzw)[*12*](https://paperpile.com/c/fJySTi/ZEXRx+dEgzw)[)](https://paperpile.com/c/fJySTi/ZEXRx+dEgzw) in our cohort across IGK or IGL, and comparing the gene usage correlations between groups of individuals with higher and lower ASDs. Repertoire-wide gene usage correlations between samples were calculated using the Pearson’s Correlation coefficient. Using all guQTL variants for each gene, individuals with the most overlapping guQTL genotypes (low ASD) had a higher mean gene usage correlation than those in the group with the highest ASD scores for both IGK (0.983 vs. 0.966; KS test *p* = 4.4e-141) and IGL (0.925 vs. 0.900 ; KS test *p* = 7.1e-03) (**Supplementary Figure 10**). These results indicated that genetic background makes a contribution to the overall gene usage composition of the repertoire, and expand on observations from twin studies [(](https://paperpile.com/c/fJySTi/fAZZE+7OQpo+AQIqn)[*7*](https://paperpile.com/c/fJySTi/fAZZE+7OQpo+AQIqn)[–](https://paperpile.com/c/fJySTi/fAZZE+7OQpo+AQIqn)[*9*](https://paperpile.com/c/fJySTi/fAZZE+7OQpo+AQIqn)[)](https://paperpile.com/c/fJySTi/fAZZE+7OQpo+AQIqn) by demonstrating that heritable components of the light chain repertoire can be directly linked to germline variants in light chain loci.

**Variants associated with IGL gene usage variation are enriched in regulatory regions**

Large-scale studies utilizing expression, epigenomic, and variant datasets associated with diseases or traits have uncovered non-coding variants within regulatory elements linked to specific phenotypes [(](https://paperpile.com/c/fJySTi/y4dnf+XjHhO+cq0CY+XtmVi)[*13*](https://paperpile.com/c/fJySTi/y4dnf+XjHhO+cq0CY+XtmVi)[–](https://paperpile.com/c/fJySTi/y4dnf+XjHhO+cq0CY+XtmVi)[*16*](https://paperpile.com/c/fJySTi/y4dnf+XjHhO+cq0CY+XtmVi)[)](https://paperpile.com/c/fJySTi/y4dnf+XjHhO+cq0CY+XtmVi). In the context of V(D)J recombination, RSS are recognized by RAG1/RAG2 proteins to direct double-strand DNA breaks and initiate somatic recombination [(](https://paperpile.com/c/fJySTi/bFMjZ)[*17*](https://paperpile.com/c/fJySTi/bFMjZ)[)](https://paperpile.com/c/fJySTi/bFMjZ). This process is regulated by cis-elements that interact with chromatin-binding proteins which collectively determine the probability of somatic recombination for a given gene [(](https://paperpile.com/c/fJySTi/qzK7p+72YJu+pJxpI+8pMPq+rZGdF)[*18*](https://paperpile.com/c/fJySTi/qzK7p+72YJu+pJxpI+8pMPq+rZGdF)[–](https://paperpile.com/c/fJySTi/qzK7p+72YJu+pJxpI+8pMPq+rZGdF)[*22*](https://paperpile.com/c/fJySTi/qzK7p+72YJu+pJxpI+8pMPq+rZGdF)[)](https://paperpile.com/c/fJySTi/qzK7p+72YJu+pJxpI+8pMPq+rZGdF). Given that non-coding variants associated with IGH gene usage variation are enriched in regulatory regions, we hypothesized that non-coding variants might impact light-chain gene usage by affecting regulatory elements. 

We tested for enrichment of candidate transcription factor binding sites (TFBS, ENCODE3 Transcription Factor ChIP-seq dataset) in IGL (302 regulatory elements) and IGK (129 regulatory elements) overlapping lead guQTL variants in IGL (97 lead SNVs) and IGK (125 lead SNVs) versus the remainder of common SNVs for each locus, which included 1017 and 2310 non-lead variants in IGL and IGK, respectively. We found that lead IGL guQTLs were enriched with TFBS for multiple factors, including SRF, CBX1, TRIM28, TAL1, EGR1, and HNRNPUL1 (**Supplementary Figure 12**). The guQTL variants overlapping these TFBS were associated with usage of IGLV3-10, IGLV2-8, IGLV3-9, IGLV3-27, IGLV2-11, and IGLV9-49 (**Supplementary Table S8**). In contrast with IGL, there was not a significant enrichment ead IGK guQTL variants within TFBS.

The lead variant associated with the usage of IGLV3-10, IGLV2-8, and IGLV3-9 (**Supplementary Figure12**) overlaps an EGR1 site in addition to >10 distinct annotated TFBS including SMC3, YY1, ETS1, ATF1, CTCF, and RAD21. Similarly, the lead variant associated with IGLV9-49 usage overlaps CBX1 and TRIM28 sites in additional to sites for >10 additional TF’s, among which were SMC3, CREB1, MYC, JUNB, STAT5A, CTCF, and RAD21 (**Supplementary Figure 13**). We noted that the IGLV9-49 lead variant is 50 bp upstream of the IGLV9-49 5’ UTR. 

These data suggest that a subset of IGL guQTLs are within cis-elements that may regulate V(D)J recombination and therefore gene usage in the peripheral antibody repertoire.  

**Genetic variants disrupt biases in differential usage of IGKV proximal and distal paralogs** 

IGKV proximal and distal gene paralogs vary substantially with respect to distance from the IGKJ region along the linear genome, with at least 1.0 Mbp separating distal V genes from J segments [(](https://paperpile.com/c/fJySTi/wG1qk)[*23*](https://paperpile.com/c/fJySTi/wG1qk)[)](https://paperpile.com/c/fJySTi/wG1qk). To date, there has not been an empirical evaluation of IGKV proximal versus distal gene usage. Historically, usage of IGKV genes has been difficult to assess due to high sequence similarity between paralogs [(](https://paperpile.com/c/fJySTi/e05pw+ask1b)[*24*](https://paperpile.com/c/fJySTi/e05pw+ask1b)[, ](https://paperpile.com/c/fJySTi/e05pw+ask1b)[*25*](https://paperpile.com/c/fJySTi/e05pw+ask1b)[)](https://paperpile.com/c/fJySTi/e05pw+ask1b). With paired AIRR-seq and germline alleles available for the first time at population-scale, we determined the individual usage of 13 IGKV paralog pairs and observed higher usage of the proximal gene in 11 cases, with the exception of IGKV2-29/2-29, for which usage was not significantly different, and IGKV1-13/1D-13, for which distal paralog usage was higher (paired t-tests, *P* value < 1.0e-10) (**Supplementary Figure 14A**). 

IGKV1-13 was used at a lower frequency than IGKV1D-13 in all but one individual. Notably, all IGKV1-13 alleles have a non-canonical heptamer (CATAGTG) (**Supplementary Figure 14B**), and IGKV1-13*01, the most frequent allele (frequency 73%, **Supplementary Figure 14C**) encodes a premature STOP codon (**Supplementary Figure 15**). Therefore, IGKV1-13 resembles a pseudogene, with both regulatory and coding loss-of-function features across haplotypes. IGKV1D-13 sequences, in contrast, included an allele (IGKV1D-13*02) with a canonical heptamer (CACAGTG) identified at a frequency of 23.6% (**Supplementary Figure 14C**). The lead IGKV1D-13 guQTL tagged this heptamer variation, as all individuals in the higher-usage C/C genotype group were also IGKV1D-13*02/IGKV1D-13*02, whereas 69 of 70 individuals in the T/T genotype group did not carry the *02 allele (**Supplementary Figure 14D**), implicating heptamer variation as a mechanism underlying usage variation.    

IGKV2-29 was used at a lower frequency than IGKV2D-29 in 114 out of 170 individuals (67.1%). This inter-individual variation in IGKV2-29 versus IGKV2D-29 usage was explained by the lead IGKV2-29 guQTL, with 109 out of 110 individuals homozygous for the reference allele having higher IGKV2D-29 usage and 15 out of 15 individuals homozygous for the alternate allele having higher IGKV2-29 usage (**Supplementary Figure 14F**). Mechanistically, the lead IGKV2-29 guQTL results in a premature STOP codon in the *01, *01_N1, and *01_N2 alleles (**Figure 2B**), which are the only IGKV2-29 alleles carried by the 110 individuals homozygous for the guQTL reference allele. 

**References**

1.	 [O. L. Rodriguez, W. S. Gibson, T. Parks, M. Emery, J. Powell, M. Strahl, G. Deikus, K. Auckland, E. E. Eichler, W. A. Marasco, R. Sebra, A. J. Sharp, M. L. Smith, A. Bashir, C. T. Watson, A Novel Framework for Characterizing Genomic Haplotype Diversity in the Human Immunoglobulin Heavy Chain Locus. ](http://paperpile.com/b/fJySTi/R4hl)[*Front. Immunol.*](http://paperpile.com/b/fJySTi/R4hl)[ ](http://paperpile.com/b/fJySTi/R4hl)[**11**](http://paperpile.com/b/fJySTi/R4hl)[, 2136 (2020).](http://paperpile.com/b/fJySTi/R4hl)

2.	 [E. Engelbrecht, O. L. Rodriguez, K. Shields, S. Schultze, D. Tieri, U. Jana, G. Yaari, W. D. Lees, M. L. Smith, C. T. Watson, Resolving haplotype variation and complex genetic architecture in the human immunoglobulin kappa chain locus in individuals of diverse ancestry. ](http://paperpile.com/b/fJySTi/7XJkC)[*Genes Immun.*](http://paperpile.com/b/fJySTi/7XJkC)[, doi: ](http://paperpile.com/b/fJySTi/7XJkC)[10.1038/s41435-024-00279-2](http://dx.doi.org/10.1038/s41435-024-00279-2)[ (2024).](http://paperpile.com/b/fJySTi/7XJkC)

3.	 [W. S. Gibson, O. L. Rodriguez, K. Shields, C. A. Silver, A. Dorgham, M. Emery, G. Deikus, R. Sebra, E. E. Eichler, A. Bashir, M. L. Smith, C. T. Watson, Characterization of the immunoglobulin lambda chain locus from diverse populations reveals extensive genetic variation. ](http://paperpile.com/b/fJySTi/RgYut)[*Genes Immun.*](http://paperpile.com/b/fJySTi/RgYut)[ ](http://paperpile.com/b/fJySTi/RgYut)[**24**](http://paperpile.com/b/fJySTi/RgYut)[, 21–31 (2023).](http://paperpile.com/b/fJySTi/RgYut)

4.	 [G. Schaible, G. A. Rappold, W. Pargent, H. G. Zachau, The immunoglobulin kappa locus: polymorphism and haplotypes of Caucasoid and non-Caucasoid individuals. ](http://paperpile.com/b/fJySTi/Z3QuC)[*Hum. Genet.*](http://paperpile.com/b/fJySTi/Z3QuC)[ ](http://paperpile.com/b/fJySTi/Z3QuC)[**91**](http://paperpile.com/b/fJySTi/Z3QuC)[, 261–267 (1993).](http://paperpile.com/b/fJySTi/Z3QuC)

5.	 [W. Pargent, K. F. Schäble, H. G. Zachau, Polymorphisms and haplotypes in the human immunoglobulin kappa locus. ](http://paperpile.com/b/fJySTi/O69c9)[*Eur. J. Immunol.*](http://paperpile.com/b/fJySTi/O69c9)[ ](http://paperpile.com/b/fJySTi/O69c9)[**21**](http://paperpile.com/b/fJySTi/O69c9)[, 1829–1835 (1991).](http://paperpile.com/b/fJySTi/O69c9)

6.	 [P. H. Sudmant, T. Rausch, E. J. Gardner, R. E. Handsaker, A. Abyzov, J. Huddleston, Y. Zhang, K. Ye, G. Jun, M. H.-Y. Fritz, M. K. Konkel, A. Malhotra, A. M. Stütz, X. Shi, F. P. Casale, J. Chen, F. Hormozdiari, G. Dayama, K. Chen, M. Malig, M. J. P. Chaisson, K. Walter, S. Meiers, S. Kashin, E. Garrison, A. Auton, H. Y. K. Lam, X. J. Mu, C. Alkan, D. Antaki, T. Bae, E. Cerveira, P. Chines, Z. Chong, L. Clarke, E. Dal, L. Ding, S. Emery, X. Fan, M. Gujral, F. Kahveci, J. M. Kidd, Y. Kong, E.-W. Lameijer, S. McCarthy, P. Flicek, R. A. Gibbs, G. Marth, C. E. Mason, A. Menelaou, D. M. Muzny, B. J. Nelson, A. Noor, N. F. Parrish, M. Pendleton, A. Quitadamo, B. Raeder, E. E. Schadt, M. Romanovitch, A. Schlattl, R. Sebra, A. A. Shabalin, A. Untergasser, J. A. Walker, M. Wang, F. Yu, C. Zhang, J. Zhang, X. Zheng-Bradley, W. Zhou, T. Zichner, J. Sebat, M. A. Batzer, S. A. McCarroll, 1000 Genomes Project Consortium, R. E. Mills, M. B. Gerstein, A. Bashir, O. Stegle, S. E. Devine, C. Lee, E. E. Eichler, J. O. Korbel, An integrated map of structural variation in 2,504 human genomes. ](http://paperpile.com/b/fJySTi/oZBB1)[*Nature*](http://paperpile.com/b/fJySTi/oZBB1)[ ](http://paperpile.com/b/fJySTi/oZBB1)[**526**](http://paperpile.com/b/fJySTi/oZBB1)[, 75–81 (2015).](http://paperpile.com/b/fJySTi/oZBB1)

7.	 [J. Glanville, T. C. Kuo, H.-C. von Büdingen, L. Guey, J. Berka, P. D. Sundar, G. Huerta, G. R. Mehta, J. R. Oksenberg, S. L. Hauser, D. R. Cox, A. Rajpal, J. Pons, Naive antibody gene-segment frequencies are heritable and unaltered by chronic lymphocyte ablation. ](http://paperpile.com/b/fJySTi/fAZZE)[*Proc. Natl. Acad. Sci. U. S. A.*](http://paperpile.com/b/fJySTi/fAZZE)[ ](http://paperpile.com/b/fJySTi/fAZZE)[**108**](http://paperpile.com/b/fJySTi/fAZZE)[, 20066–20071 (2011).](http://paperpile.com/b/fJySTi/fAZZE)

8.	 [F. Rubelt, C. R. Bolen, H. M. McGuire, J. A. Vander Heiden, D. Gadala-Maria, M. Levin, G. M. Euskirchen, M. R. Mamedov, G. E. Swan, C. L. Dekker, L. G. Cowell, S. H. Kleinstein, M. M. Davis, Individual heritable differences result in unique cell lymphocyte receptor repertoires of naïve and antigen-experienced cells. ](http://paperpile.com/b/fJySTi/7OQpo)[*Nat. Commun.*](http://paperpile.com/b/fJySTi/7OQpo)[ ](http://paperpile.com/b/fJySTi/7OQpo)[**7**](http://paperpile.com/b/fJySTi/7OQpo)[, 11112 (2016).](http://paperpile.com/b/fJySTi/7OQpo)

9.	 [C. Wang, Y. Liu, M. M. Cavanagh, S. Le Saux, Q. Qi, K. M. Roskin, T. J. Looney, J.-Y. Lee, V. Dixit, C. L. Dekker, G. E. Swan, J. J. Goronzy, S. D. Boyd, B-cell repertoire responses to varicella-zoster vaccination in human identical twins. ](http://paperpile.com/b/fJySTi/AQIqn)[*Proc. Natl. Acad. Sci. U. S. A.*](http://paperpile.com/b/fJySTi/AQIqn)[ ](http://paperpile.com/b/fJySTi/AQIqn)[**112**](http://paperpile.com/b/fJySTi/AQIqn)[, 500–505 (2015).](http://paperpile.com/b/fJySTi/AQIqn)

10.	 [O. L. Rodriguez, Y. Safonova, C. A. Silver, K. Shields, W. S. Gibson, J. T. Kos, D. Tieri, H. Ke, K. J. L. Jackson, S. D. Boyd, M. L. Smith, W. A. Marasco, C. T. Watson, Genetic variation in the immunoglobulin heavy chain locus shapes the human antibody repertoire. ](http://paperpile.com/b/fJySTi/r6wFt)[*Nat. Commun.*](http://paperpile.com/b/fJySTi/r6wFt)[ ](http://paperpile.com/b/fJySTi/r6wFt)[**14**](http://paperpile.com/b/fJySTi/r6wFt)[, 4419 (2023).](http://paperpile.com/b/fJySTi/r6wFt)

11.	 [X. Gao, E. R. Martin, Using allele sharing distance for detecting human population stratification. ](http://paperpile.com/b/fJySTi/ZEXRx)[*Hum. Hered.*](http://paperpile.com/b/fJySTi/ZEXRx)[ ](http://paperpile.com/b/fJySTi/ZEXRx)[**68**](http://paperpile.com/b/fJySTi/ZEXRx)[, 182–191 (2009).](http://paperpile.com/b/fJySTi/ZEXRx)

12.	 [X. Gao, J. Starmer, Human population structure detection via multilocus genotype clustering. ](http://paperpile.com/b/fJySTi/dEgzw)[*BMC Genet.*](http://paperpile.com/b/fJySTi/dEgzw)[ ](http://paperpile.com/b/fJySTi/dEgzw)[**8**](http://paperpile.com/b/fJySTi/dEgzw)[, 34 (2007).](http://paperpile.com/b/fJySTi/dEgzw)

13.	 [GTEx Consortium, The GTEx Consortium atlas of genetic regulatory effects across human tissues. ](http://paperpile.com/b/fJySTi/y4dnf)[*Science*](http://paperpile.com/b/fJySTi/y4dnf)[ ](http://paperpile.com/b/fJySTi/y4dnf)[**369**](http://paperpile.com/b/fJySTi/y4dnf)[, 1318–1330 (2020).](http://paperpile.com/b/fJySTi/y4dnf)

14.	 [C. A. Boix, B. T. James, Y. P. Park, W. Meuleman, M. Kellis, Regulatory genomic circuitry of human disease loci by integrative epigenomics. ](http://paperpile.com/b/fJySTi/XjHhO)[*Nature*](http://paperpile.com/b/fJySTi/XjHhO)[ ](http://paperpile.com/b/fJySTi/XjHhO)[**590**](http://paperpile.com/b/fJySTi/XjHhO)[, 300–307 (2021).](http://paperpile.com/b/fJySTi/XjHhO)

15.	 [Roadmap Epigenomics Consortium et al. Integrative analysis of 111 reference human epigenomes. ](http://paperpile.com/b/fJySTi/cq0CY)[*Nature*](http://paperpile.com/b/fJySTi/cq0CY)[.](http://paperpile.com/b/fJySTi/cq0CY)

16.	 [K. K.-H. Farh, A. Marson, J. Zhu, M. Kleinewietfeld, W. J. Housley, S. Beik, N. Shoresh, H. Whitton, R. J. H. Ryan, A. A. Shishkin, M. Hatan, M. J. Carrasco-Alfonso, D. Mayer, C. J. Luckey, N. A. Patsopoulos, P. L. De Jager, V. K. Kuchroo, C. B. Epstein, M. J. Daly, D. A. Hafler, B. E. Bernstein, Genetic and epigenetic fine mapping of causal autoimmune disease variants. ](http://paperpile.com/b/fJySTi/XtmVi)[*Nature*](http://paperpile.com/b/fJySTi/XtmVi)[ ](http://paperpile.com/b/fJySTi/XtmVi)[**518**](http://paperpile.com/b/fJySTi/XtmVi)[, 337–343 (2015).](http://paperpile.com/b/fJySTi/XtmVi)

17.	 [S. D. Fugmann, A. I. Lee, P. E. Shockett, I. J. Villey, D. G. Schatz, The RAG proteins and V(D)J recombination: complexes, ends, and transposition. ](http://paperpile.com/b/fJySTi/bFMjZ)[*Annu. Rev. Immunol.*](http://paperpile.com/b/fJySTi/bFMjZ)[ ](http://paperpile.com/b/fJySTi/bFMjZ)[**18**](http://paperpile.com/b/fJySTi/bFMjZ)[, 495–527 (2000).](http://paperpile.com/b/fJySTi/bFMjZ)

18.	 [L. Hill, G. Wutz, M. Jaritz, H. Tagoh, L. Calderón, J.-M. Peters, A. Goloborodko, M. Busslinger, Igh and Igk loci use different folding principles for V gene recombination due to distinct chromosomal architectures of pro-B and pre-B cells. ](http://paperpile.com/b/fJySTi/qzK7p)[*Nat. Commun.*](http://paperpile.com/b/fJySTi/qzK7p)[ ](http://paperpile.com/b/fJySTi/qzK7p)[**14**](http://paperpile.com/b/fJySTi/qzK7p)[, 2316 (2023).](http://paperpile.com/b/fJySTi/qzK7p)

19.	 [K. H. Bhat, S. Priyadarshi, S. Naiyer, X. Qu, H. Farooq, E. Kleiman, J. Xu, X. Lei, J. F. Cantillo, R. Wuerffel, N. Baumgarth, J. Liang, A. J. Feeney, A. L. Kenter, An Igh distal enhancer modulates antigen receptor diversity by determining locus conformation. ](http://paperpile.com/b/fJySTi/72YJu)[*Nat. Commun.*](http://paperpile.com/b/fJySTi/72YJu)[ ](http://paperpile.com/b/fJySTi/72YJu)[**14**](http://paperpile.com/b/fJySTi/72YJu)[, 1225 (2023).](http://paperpile.com/b/fJySTi/72YJu)

20.	 [E. M. Barajas-Mora, L. Lee, H. Lu, J. A. Valderrama, E. Bjanes, V. Nizet, A. J. Feeney, M. Hu, C. Murre, Enhancer-instructed epigenetic landscape and chromatin compartmentalization dictate a primary antibody repertoire protective against specific bacterial pathogens. ](http://paperpile.com/b/fJySTi/pJxpI)[*Nat. Immunol.*](http://paperpile.com/b/fJySTi/pJxpI)[ ](http://paperpile.com/b/fJySTi/pJxpI)[**24**](http://paperpile.com/b/fJySTi/pJxpI)[, 320–336 (2023).](http://paperpile.com/b/fJySTi/pJxpI)

21.	 [D. J. Bolland, H. Koohy, A. L. Wood, L. S. Matheson, F. Krueger, M. J. T. Stubbington, A. Baizan-Edge, P. Chovanec, B. A. Stubbs, K. Tabbada, S. R. Andrews, M. Spivakov, A. E. Corcoran, Two mutually exclusive local chromatin states drive efficient V(D)J recombination. ](http://paperpile.com/b/fJySTi/8pMPq)[*Cell Rep.*](http://paperpile.com/b/fJySTi/8pMPq)[ ](http://paperpile.com/b/fJySTi/8pMPq)[**15**](http://paperpile.com/b/fJySTi/8pMPq)[, 2475–2487 (2016).](http://paperpile.com/b/fJySTi/8pMPq)

22.	 [D. G. Schatz, Y. Zhang, J. Xiao, S. Zha, Y. Zhang, F. W. Alt, “The mechanism, regulation and evolution of V(D)J recombination” in ](http://paperpile.com/b/fJySTi/rZGdF)[*Molecular Biology of B Cells*](http://paperpile.com/b/fJySTi/rZGdF)[, T. Honjo, M. Reth, A. Radbruch, F. Alt, A. Martin, Eds. (Elsevier, 2024), pp. 13–57.](http://paperpile.com/b/fJySTi/rZGdF)

23.	 [K. Kawasaki, S. Minoshima, E. Nakato, K. Shibuya, A. Shintani, S. Asakawa, T. Sasaki, H. G. Klobeck, G. Combriato, H. G. Zachau, N. Shimizu, Evolutionary dynamics of the human immunoglobulin kappa locus and the germline repertoire of the Vkappa genes. ](http://paperpile.com/b/fJySTi/wG1qk)[*Eur. J. Immunol.*](http://paperpile.com/b/fJySTi/wG1qk)[ ](http://paperpile.com/b/fJySTi/wG1qk)[**31**](http://paperpile.com/b/fJySTi/wG1qk)[, 1017–1028 (2001).](http://paperpile.com/b/fJySTi/wG1qk)

24.	 [A. M. Collins, M. Ohlin, M. Corcoran, J. M. Heather, D. Ralph, M. Law, J. Martínez-Barnetche, J. Ye, E. Richardson, W. S. Gibson, O. L. Rodriguez, A. Peres, G. Yaari, C. T. Watson, W. D. Lees, AIRR-C IG Reference Sets: curated sets of immunoglobulin heavy and light chain germline genes. ](http://paperpile.com/b/fJySTi/e05pw)[*Front. Immunol.*](http://paperpile.com/b/fJySTi/e05pw)[ ](http://paperpile.com/b/fJySTi/e05pw)[**14**](http://paperpile.com/b/fJySTi/e05pw)[, 1330153 (2023).](http://paperpile.com/b/fJySTi/e05pw)

25.	 [I. Mikocziova, A. Peres, M. Gidoni, V. Greiff, G. Yaari, L. M. Sollid, Germline polymorphisms and alternative splicing of human immunoglobulin light chain genes. ](http://paperpile.com/b/fJySTi/ask1b)[*iScience*](http://paperpile.com/b/fJySTi/ask1b)[ ](http://paperpile.com/b/fJySTi/ask1b)[**24**](http://paperpile.com/b/fJySTi/ask1b)[, 103192 (2021).](http://paperpile.com/b/fJySTi/ask1b)
